# Binocularly suppressed stimuli induce brain activities related to aesthetic emotions

**DOI:** 10.3389/fnins.2024.1339479

**Published:** 2024-05-24

**Authors:** Hideyuki Hoshi, Akira Ishii, Yoshihito Shigihara, Takahiro Yoshikawa

**Affiliations:** ^1^Department of Sports Medicine, Osaka Metropolitan University Graduate School of Medicine, Osaka, Japan; ^2^Precision Medicine Centre, Hokuto Hospital, Obihiro, Japan

**Keywords:** magnetoencephalography, vision, continuous flash suppression, neuroaesthetics, aesthetic emotion

## Abstract

**Introduction:**

Aesthetic emotions are a class of emotions aroused by evaluating aesthetically appealing objects or events. While evolutionary aesthetics suggests the adaptive roles of these emotions, empirical assessments are lacking. Previous neuroscientific studies have demonstrated that visual stimuli carrying evolutionarily important information induce neural responses even when presented non-consciously. To examine the evolutionary importance of aesthetic emotions, we conducted a neuroscientific study using magnetoencephalography (MEG) to measure induced neural responses to non-consciously presented portrait paintings categorised as biological and non-biological and examined associations between the induced responses and aesthetic ratings.

**Methods:**

MEG and pre-rating data were collected from 23 participants. The pre-rating included visual analogue scales for *object saliency*, *facial saliency*, *liking*, and *beauty* scores, in addition to ‘*biologi-ness*,’ which was used for subcategorising stimuli into biological and non-biological. The stimuli were presented non-consciously using a continuous flash suppression paradigm or consciously using binocular presentation without flashing masks, while dichotomic behavioural responses were obtained (beauty or non-beauty). Time-frequency decomposed MEG data were used for correlation analysis with pre-rating scores for each category.

**Results:**

Behavioural data revealed that saliency scores of non-consciously presented stimuli influenced dichotomic responses (beauty or non-beauty). MEG data showed that non-consciously presented portrait paintings induced spatiotemporally distributed low-frequency brain activities associated with aesthetic ratings, which were distinct between the biological and non-biological categories and conscious and non-conscious conditions.

**Conclusion:**

Aesthetic emotion holds evolutionary significance for humans. Neural pathways are sensitive to visual images that arouse aesthetic emotion in distinct ways for biological and non-biological categories, which are further influenced by consciousness. These differences likely reflect the diversity in mechanisms of aesthetic processing, such as processing fluency, active elaboration, and predictive processing. The aesthetic processing of non-conscious stimuli appears to be characterised by fluency-driven affective processing, while top-down regulatory processes are suppressed. This study provides the first empirical evidence supporting the evolutionary significance of aesthetic processing.

## 1 Introduction

Aesthetic processing is a complex human function encompassing psychological processes, including perceptual, cognitive, and emotional evaluations of aesthetically appealing information, leading to emotional and hedonic experiences. Emotional reactions within these processes are specifically referred to as ‘aesthetic emotions,’ aroused in response to the aesthetic qualities of the stimulus, and are predictive of aesthetic pleasure or displeasure and approach–avoidance behaviour. Examples include ‘feeling of beauty,’ ‘being moved,’ and ‘experiencing suspense’ (Wassiliwizky and Menninghaus, 2021). Humans are assumed to have evolved to aesthetically evaluate the sensory and perceptual qualities of objects, a concept extensively discussed in the field of Darwinian aesthetics (Grammer et al., 2003) or evolutionary aesthetics (Voland and Grammer, 2003). Therefore, the neural processes underlying aesthetic emotions are considered to be evolutionarily grounded and strongly registered functions of the human brain.

Evolutionary aesthetics investigates aesthetic preferences for sensory inputs that facilitate selective attention and emotional responses to perceived objects, leading to adaptive decision-making and problem-solving. Evolutionary aesthetics considers any object as a sensory input, ranging from biological (such as faces, bodies, movements, and vocalisations) to artefactual/cultural objects and events (such as artworks, ornaments, and artful decorations) (Voland and Grammer, 2003). Aesthetic emotions toward biological objects have been associated with mate choice decisions, with the arousal of such emotions contributing to more optimal selections and, thus, fitness benefits. Darwin first introduced the concept of ‘sexual selection’ in ‘The Descent of Man’ (Darwin, 1871), explaining how females in the species prefer certain traits in males, typically features that signify health, superiority, and strength over others, which indicate increased probability of successful reproduction. Size, colour, shape, voice, and smell serve as representative indices of sexual selection. For instance, a ‘good’ body proportion and facial symmetry are common aesthetically favoured traits used by humans during mate selection, as they are indicative of good genotypes (Thornhill et al., 1999; Grammer et al., 2003; Rhodes, 2005; Little et al., 2011; Chatterjee, 2014). Although cultural and individual differences exist, balanced faces and well-proportioned bodies are generally recognised as ‘beautiful’ and are considered attractive when encountered. This finding indicates that the aesthetic emotion aroused by biological objects originated as a physiological reaction in response to important information related to mate selection; therefore, it can be considered congenital. Several studies have suggested that new-borns and infants exhibit strong preferences for symmetrical and attractive faces (Bornstein et al., 1981; Langlois et al., 1987; Grammer et al., 2003; Rhodes, 2005). This implies that the neural system governing the aesthetic emotion aroused by biological objects is registered at a genetic level and has been shaped by natural selection.

When considering non-biological artefactual objects or events as sources of aesthetic emotion, the evolutionary explanation becomes more complicated. Anthropological studies have suggested that the creation of artworks or decorations is a defining feature of the human species, alongside the use of symbolic communication systems, the formation of societies, and the manufacture of complex tools (Mcbrearty and Brooks, 2000; d’Errico and Stringer, 2011). While this explains the evolutionary background of creation, it does not capture the appreciation (including evaluation and subsequent emotional reactions and judgements) of artistic activities. Some researchers have proposed that the aesthetic appreciation of artefactual objects is merely a by-product of adaptation and lacks evolutionary importance (Pinker, 1997; Carroll, 1998; Pinker and Fodor, 2005). In contrast, evolutionary aesthetics provides adaptive explanations regarding the aesthetic emotion aroused by artefactual objects, distinct from that of biological objects. For example, the creation and appreciation of artful objects can be likened to a specific animal behaviour known as ‘display,’ which is often performed by males to attract females, who, in turn, evolve to evaluate male displays to attract the best mate (Etcoff, 1999; Rothenberg, 2011). Similarly, artistic communication can be linked to the behaviour of ‘gift’ in some species, such as providing food or nests, or displaying features such as agility, strength, or hunting skills, all of which are useful for reproduction and evaluated by females (Voland and Grammer, 2003). Moreover, cultural neuroscience research suggests that human cultural differences shape our neural systems, from basic perceptual systems to higher-order systems such as social cognition, as highlighted by the quote ‘culture is, after all, stored in people’s brains’ (Ames and Fiske, 2010), which implies a congenital factor in the affective responses to cultural objects. Taken together, supportive views regarding the evolutionary significance of the aesthetic emotion aroused by non-biological objects appear to be more dominant, despite lacking scientific support. An empirical study examining the qualitative differences in the links to evolutionary significance between biological and artefactual objects demonstrated that the magnitude of aesthetic emotion aroused by biological objects was less likely to be influenced by external opinions compared to that of artefactual objects (Bignardi et al., 2021). The aesthetic ratings for natural objects (faces and landscapes) have also shown stronger inter- and intra-individual agreement than those of artefactual objects (artworks and architectures) (Vessel et al., 2014, 2018), suggesting that shared characteristics of biological stimuli arouse aesthetic emotion. These findings correspond with the evolutionary implication that aesthetic emotions aroused by biological objects are strongly ingrained at the genetic level. Furthermore, these studies indicate that objects recognised as ‘more biological’ and ‘less biological’ have distinct evolutionary relevance. Therefore, investigating both categories separately is warranted when determining the evolutionary importance of aesthetic emotion.

To establish a comprehensive evolutionary explanation of an adaptive trait, its proximate mechanisms (including neural circuitry and emotions controlling mental representations and behaviour) must be clarified (Tinbergen, 1963; Rusch and Voland, 2013). From a neuroscientific perspective, the evolutionary significance of the emotional processing of biological objects has been examined using the subliminal presentation of emotionally arousing faces (Brooks et al., 2012; Dahlén et al., 2022). Studies have demonstrated that arousing faces activate brain regions responsive to emotional processing, even when presented non-consciously or without observer awareness, such as when presented very briefly and immediately masked (masking paradigm), suppressed binocularly (such as continuous flash suppression: CFS paradigm), or under other experimental conditions (Axelrod et al., 2015). Similar findings have been observed for non-biological stimulus categories comparable to emotional faces, such as spiders, snakes, guns, and valenced words (Carlsson et al., 2004; Wendt et al., 2008; Alpers et al., 2009; van Gaal et al., 2014; Fang et al., 2016; Mudrik and Deouell, 2022). High sensitivity and responsiveness to emotional stimuli are directly linked to evolutionary importance because these characteristics allow individuals to quickly respond to approaching harm, thereby enhancing the chance of survival. Non-conscious studies have indicated that the neural processing system is tuned to rapidly detect signals of approaching harm with great sensitivity to avoid threats. Further evidence supports the idea that neural processing systems are tuned for both avoidance and approach. For example, neural responses were recorded for non-consciously presented positive emotions (such as happiness and surprise) and words (Killgore and Yurgelun-Todd, 2004; Somerville et al., 2004; Sergerie et al., 2008; Brooks et al., 2012; van Gaal et al., 2014; Dahlén et al., 2022). A series of neuroscientific findings suggest that the neural system is designed to be responsive to evolutionarily significant information presented non-consciously through any stimulus category (biological or non-biological). Notably, the specialised neural system for processing this evolutionarily important information is not entirely universal or ‘hard-coded’; it may be congenital but can also be shaped by environmental factors. Although the neural system is strongly attuned to certain basic visual features of biological stimuli, such as ‘good’ body proportions and facial symmetry, which are universally recognised across individuals, individual-level fine-tuning remains possible. For example, the ‘other-race effect’ (Stelter and Schweinberger, 2023) explains the phenomenon where facial stimuli belonging to the same ethnic group as the viewer induce distinct autonomic neural responses compared to those from other ethnic groups (Pesciarelli et al., 2021). Facial familiarity also produces similar effects (Axelrod et al., 2015). These studies indicate individual differences in the processing of ‘biological’ visual features that are connected to their evolutionary significance. Therefore, we operationally define the biological and non-biological categories as discrete conversions of a continuous level of ‘*biologi-ness*’, reflecting the individual’s level of experience in considering an object as biological, based on their intuitive feeling, prior knowledge, and/or experiences.

In this study, we aimed to investigate whether aesthetic emotion has an adaptive function in humans by examining neural responses to aesthetically appealing biological and non-biological objects presented non-consciously. To achieve non-conscious presentation, we used CFS, which allowed for a longer duration of presentation (unlike the masking paradigm) without manipulating the colours of the stimuli (unlike the binocular fusion/rivalry paradigms) (Tsuchiya and Koch, 2004, 2005). Although the precise neural mechanisms underlying the processing of non-consciously presented emotional stimuli remain unclear, a subcortical pathway, which bypasses cortical input and rapidly conveys emotional information to the emotional brain, has been reported to potentially characterise non-conscious processing (LeDoux, 1996, but see Pessoa and Adolphs, 2010). To capture the temporal dynamics with good spatial resolution (Baillet, 2017; Hari and Puce, 2017), we used magnetoencephalography (MEG) to measure neural responses during the CFS task. We hypothesised that if aesthetic emotion has adaptive significance, non-consciously presented aesthetically appealing stimuli should induce MEG responses related to emotional processing. To the best of our knowledge, this is the first neuroscientific study to reveal the evolutionary significance of aesthetic emotions.

## 2 Materials and methods

### 2.1 Participants and ethics

This study involved 26 healthy adult Japanese participants with no history of neurological or psychiatric disorders or professional education in artistic disciplines. Three participants were excluded from the analysis: one due to unremovable artefacts (e.g., saturated signals) in the recorded MEG data from previous dental work, and two for failure to maintain dichoptic vision, leading to ineffective stimulus suppression with CFS. Data obtained from the remaining 23 participants (16 females, all right-handed, mean age 35.74 ± 10.35 years) were analysed. This study was conducted in accordance with the ethical principles of the Declaration of Helsinki and was approved by the Ethics Committee of Hokuto Hospital (approval number: #1108) and Osaka Metropolitan University Graduate School of Medicine (2022-034). Written informed consent was obtained from all participants prior to their involvement in the study.

### 2.2 Stimuli

**FIGURE 1:**
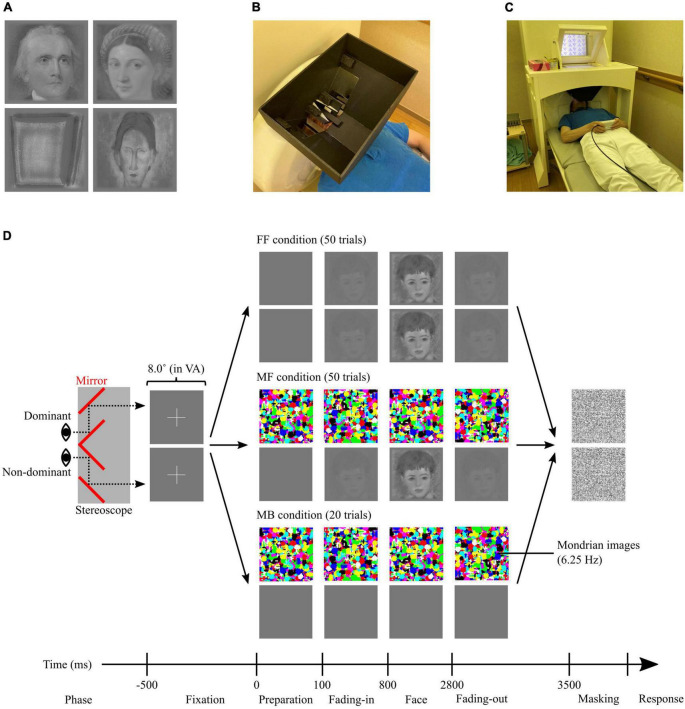
**(A)** Examples of facial stimuli after editing; (left top) the stimulus that scored the highest average ‘*biologi-ness*’ score (ID#19), (right top) the stimulus that scored the highest average *beauty* score (ID#06), (left bottom) the stimulus that scored the lowest average ‘*biologi-ness*’, *object* and *facial saliency*, and *beauty* scores (ID#48), and (right bottom) the stimulus that scored the lowest average *liking* score (ID#35). **(B)** A non-magnetic stereoscope, placed on the MEG dewer before starting the MEG experiment. **(C)** Setup for the MEG experiment showing one of our researchers laying down on the bed (instead of the participant) for the purposes of photographing. **(D)** Schematic illustration of the CFS task. Participants were presented different stimuli to the dominant and non-dominant eyes through the stereoscope. All trials started from the presentation of the fixations, then diverted according to the condition (FF, MF, and MB). FF, face-face condition; MF, Mondrian-face condition; MB, Mondrian-background condition; VA, visual angle.

A set of 5 portrait paintings by 10 artists was sourced from WikiArt^1^ (Supplementary Table 1). The paintings were selected to achieve gender balance (women, men, and unknown) among the painted models portrayed by each artist, ensuring a difference in the number of female and male models of < 2 (e.g., three women and two men). All paintings were of Western origin, spanning various styles from the 16th to the 20th centuries, and explicitly depicted human faces (portraits) or contained identifiable keywords depicting the presence of human faces, such as ‘head’ and ‘man with a moustache.’ All paintings were cropped to focus solely on the facial areas (from the top of the head to the chin) in a square of 500 × 500 pixels, converted into greyscale, and equated for spatial frequencies and histograms using the SHINE toolbox (Willenbockel et al., 2010). Finally, to enhance the suppression effect, the image edges were blurred, blending them into a background colour, defined as the mean luminance (intensity) of all images. For this process, an additional 25 pixels with the background colour were added to each side of the image, which were blurred by filtering with a two-dimensional Gaussian smoothing kernel with a σ (standard deviation of the Gaussian distribution, indicating the filtering effect strength) determined as a normal probability density function relative to the location of the pixel to the edge. Essentially, pixels closer to the edge were more strongly blurred, and vice versa, ensuring a smooth transition between the image and background colour. The facial stimuli were sorted based on the painters and their era (Supplementary Table 1), with their representative images depicted in Figure 1A.

### 2.3 Pre-ratings

Prior to the MEG experiment, participants completed pre-rating questionnaires to assess the intensity of the aesthetic emotion aroused by each facial stimulus. The paper-based questionnaire contained five questions/ratings for each stimulus, covering ‘*biologi-ness*’ (‘How much do you find the painted objects biological?’), *object saliency* (‘How well can you recognise the painted object?’), *facial saliency* (‘How well can you recognise a face in the painting?’), *liking* (‘How much do you like the painting?’), and *beauty* (‘How much beauty do you feel from the painting?’). *Object* and *facial saliency* scores were obtained to assess the intensity of perceptual and cognitive processing, an influential factor in aesthetic emotion (Reber et al., 1998, 2004). The *beauty* score was the main target of the analysis, representing a direct measure of positive aesthetic emotion, while the *liking* score was used to measure the intensity of aesthetic pleasure predicted by aesthetic emotion (Wassiliwizky and Menninghaus, 2021). Participants used a visual analogue scale (VAS) to indicate their subjective experience by drawing a vertical line on a horizontal line ranging from each extreme (‘*biologi-ness*’: from ‘not biological at all’ to ‘very biological’; *object saliency*: from ‘I cannot recognise at all’ to ‘I can recognise clearly’; *facial saliency*: from ‘I cannot recognise at all’ to ‘I can recognise clearly’; *liking*: from ‘I do not like it at all’ to ‘I like it very much’; and *beauty*: from ‘not beautiful at all’ to ‘very beautiful’). The VAS scores were normalised, that is, converted from 0 (negative extreme) to 1 (positive extreme) by measuring the position of the vertical line (response) from the edge of the horizontal line and used for the analysis. Notably, paper-based pre-rating questionnaires were used to minimise the duration of the MEG experiment and reduce potential eye fatigue among participants while maintaining dichoptic vision with stereoscope, which would lead to poor suppression effects of CFS. However, this repeated exposure of the stimuli through the use of pre-rating may have influenced the intensity of aesthetic pleasures, a limitation we have acknowledged in section 4.5. To validate the pre-rating scales, particularly for the unique measure employed in this study (‘*biologi-ness*’), three participants underwent a retest after 1 year of the initial pre-rating date (range, 409–598 days). The test-retest correlation (Spearman’s *rho*) of the ‘*biologi-ness*’ rating was comparable to that of *object* and *facial saliency* scores, indicating good reliability and replicability of the measurement (Supplementary Table 2).

### 2.4 MEG experiment: procedure, task, and apparatus

The MEG experiment was conducted within 5 days of completing the pre-ratings. First, participants received instructions about the tasks and familiarised themselves with dichoptic vision using a handmade, non-magnetic stereoscope (Figure 1B). Subsequently, the participants lay down in a supine position in a magnetically shielded room (Figure 1C), with the stereoscope installed on the MEG dewer (between the participant’s face and the screen). Through the stereoscope, participants viewed the left half of the screen with their left eye and vice versa. To achieve the best suppression effect in CFS, the positions of stimuli presented to the left and right eyes were individually adjusted whereby the participants were presented with two squares (8.0° × 8.0° in visual angle) on the screen, one for each visual field, and asked to move around and find positions where the two overlapped perfectly in subjective perception (dichoptic vision). All images and stimuli were projected onto a screen placed 28.7 cm in front of the participants from outside the magnetically shielded room using a projector (PROPIxx; VPixx Technologies, Saint-Bruno, Canada) through a mirror. MEG data were recorded during the CFS task, which comprised four sessions, each containing 30 trials. Of the 120 trials, 50 were categorised as face-face (FF), another 50 as Mondrian-face (MF), and the remaining 20 as Mondrian-background (MB). In the FF and MF conditions, 50 facial stimuli were randomly presented once for each condition. The order of the three conditions was pseudo-randomised to ensure that no more than three consecutive trials belonged to the same condition. Figure 1D depicts the differences between the conditions. In the FF condition, each trial started with the presentation of fixation (2.7° × 2.7° in visual angle; fixation phase, 500 ms). Then, the facial stimulus faded in (fading-in phase, 700 ms), remained fixed on the screen (face phase, 2,000 ms), faded out (fading-out phase, 700 ms), and its aftereffect was suppressed by presenting a noisy mask (masking phase, 500 ms). The noisy mask comprised an image filled with 10,000 greyscale dots (arranged in a 100 × 100 matrix within a square of 8.0° × 8.0° in visual angle) of random intensity. In this condition, identical content was presented to both eyes, allowing participants to clearly visualise and consciously process the content. In the MF condition, flashing Mondrian images were presented to the participant’s dominant eye instead of a facial stimulus. The Mondrian images were randomly generated images with 1,000 randomly sized ellipses coloured in one of eight primary colours: red ([255 0 0] in RGB), green ([0 255 0]), blue ([0 0 255]), cyan ([0 255 255]), magenta ([255 0 255]), yellow ([255 255 0]), black ([0 0 0]), and white ([255 255 255]). The presentation of Mondrian images commenced 100 ms before the fading-in phase (preparation phase), where the images were sequentially refreshed with a temporal frequency tuned at 6.25 Hz, as previous studies indicated peak effectiveness of the masking/suppression at approximately 6 Hz (Zhu et al., 2016; Drewes et al., 2018). Participants who successfully maintained dichoptic vision were aware of only the flashing Mondrian stimuli presented to the dominant eye and were unaware of the facial stimuli presented to the non-dominant eye. Thus, the facial stimuli were processed non-consciously in the MF condition. In the MB condition, Mondrian images were presented to the dominant eye, while a stable background-coloured square was presented to the non-dominant eye. Similar to the MF condition, participants were aware of only the flashing Mondrian stimuli and were unaware of the background-coloured image presented to the non-dominant eye.

After each trial, the participants were asked to respond to two-alternative forced choice questions: Q1: ‘Did you see a face?,’ which was asked by presenting ‘See?’ on the screen (response phase 1) and Q2: ‘Do you feel beauty from the painting?,’ which was asked by presenting ‘Beauty?’ on the screen (response phase 2). Participants indicated answers by pressing one of the two buttons corresponding to ‘yes’ and ‘no’ using their left and right thumbs, with positions counterbalanced between participants. Participants were instructed to respond ‘yes’ to the Q1 (‘see?’ question) not only when the whole facial stimulus was visible but also if any minute part of the stimulus was visible despite Mondrian suppression. Furthermore, participants were instructed to guess the answer to Q2 (‘beauty?’ question) when they did not (consciously) see any faces in the trial. Responses to Q1 were used solely for screening and rejecting trials where the facial stimuli were not perfectly masked in the MF condition. Each response phase lasted until the participants provided their responses, with an inter-trial interval set at 1 s. Each session lasted approximately 4–5 min, varying based on the response time. The entire experimental procedure, including preparation, lasted approximately 60–90 min.

### 2.5 MEG experiment: scanning details

Cortical activity during the CFS task was recorded using a 160-channel whole-head-type MEG system (MEG vision PQ1160C; Yokogawa Electric Co., Kanazawa, Japan). During the scan, participants were asked to remain calm in a supine position in a magnetically shielded room, with scanning conditions controlled for consistency and comfort. The sensor and reference coils were gradiometers of 15.5 mm in diameter and 50 mm in baseline. Each pair of sensor coils was separated by a distance of 23 mm. The sampling frequency was 2,000 Hz, with 500 Hz low-pass filtering during recording. To co-register the MEG data with the anatomical brain images, five fiducial magnetic marker coils were placed on each participant’s face (40 mm above the nasion, bilaterally 10 mm in front of the tragus, and bilateral pre-auricular points) before the MEG scan, and their spatial coordinates were measured immediately before each session. During the scan, participants were monitored by a technical staff member using a video camera installed in the magnetically shielded room. Among the 23 participants, the individual magnetic resonance imaging (MRI) data of 5 participants were obtained retrospectively from another study. These were anatomical T1-weighted MR images acquired using a 3.0-T scanner (SIGMA Excite 3.0T, GE Healthcare, Milwaukee, WI, USA) with a standard head coil and three fiducial markers (Medtronic Surgical Navigation Technologies Inc., Broomfield, CO, USA) positioned at the three magnetic marker coils placed on the forehead.

### 2.6 MEG data analysis

MEG data were analysed offline using RICOH MEG Analysis software (RICOH, Tokyo, Japan), MATLAB (MathWorks, MA, USA), Brainstorm, which is documented and freely available for download online under the GNU general public licence^2^ (Tadel et al., 2011), and the FreeSurfer image analysis suite, which is documented and freely available for download online.^3^ First, continuous MEG signals were cleaned using a dual-signal subspace projection algorithm (Sekihara et al., 2016) available in the vendor-provided software (RICOH MEG Analysis), comparable to the temporally extended signal-space separation algorithm, with the only difference being the approximation of the signal subspace projector (Cai et al., 2019). Next, to remove the remaining artefacts, the signals were decomposed via independent component analysis using the infomax algorithm implemented in Brainstorm (Makeig et al., 1996). Each component from the independent component analysis was visually inspected, and those with cardiac, blinking, and other salient artefacts were rejected. The data were divided into 6,000 ms epochs, each starting 1,500 ms before the preparation phase (1,000 ms before the fixation phase) and lasting 1,000 ms after the fading-out phase. This epoch length was designed to avoid edge effects in the time window of interest (from the preparation to the fading-out phase) caused by the filtering process for computing the envelopes, as described later. The starting point of the time window of interest (the onset of the preparation phase) was considered to be 0 ms throughout the analysis. The offset was removed using baseline signals averaged across −1,000 to 0 ms for each sensor and epoch. Epochs (trials) were rejected and excluded from the following analyses based on three criteria: (i) epochs corresponding to trials in the MF condition where participants responded ‘yes’ to the Q1 (‘see?’ question), indicating imperfect masking of facial stimuli (ii) technical issues resulting in the last trial of the session, during which the MEG measurement was finished, not being recorded, and (iii) trials contaminated with remaining artefacts. Based on these criteria, the number of trials rejected was 1.17 ± 2.48 trials (with a maximum of 9 trials) per participant, 1 trial from two participants and 2 trials from one participant, and 3.57 ± 3.07 trials (with a maximum of 10 trials) per participant, respectively. Consequently, the number of trials considered for the analyses were: FF: 48.70 ± 1.40 trials (19.48 ± 0.59 trials for biological, and 19.35 ± 1.07 trials for non-biological stimuli), MF: 47.09 ± 2.97 trials (18.91 ± 1.28 trials for biological and 18.74 ± 1.48 trials for non-biological stimuli), and MB: 19.30 ± 0.88 trials.

The remaining artefact-free signals were projected onto the cortical source using the default parameters of the Brainstorm toolbox. For the structural MRI data, ICBM152, a template anatomical brain image prepared by Brainstorm, was used for analysis. The ICBM152 is a non-linear average of 152 MR images from different subjects (Fonov et al., 2009) and is provided along with the cortical segments. In cases where individual MRI data were obtained retrospectively from another study (5 out of 23 participants), the T1-weighted MR images were segmented using the FreeSurfer pipeline ‘recon-all’ (Fischl, 2012) and then imported into Brainstorm. The signal source was restricted to the cortex, which was segmented into 15,000 vertices. Each MEG session was co-registered with the anatomical image using the spatial coordinates of five fiducial points and the nasion, and the relationship between 160 MEG channels and 15,000 vertices (leadfield matrix) was modelled (forward modelling) using an overlapping sphere model, a recommended option for MEG data in the Brainstorm. Before computing the source signals, the characteristics of the MEG sensor noise were modelled as a covariance matrix for each pair of channels (noise covariance), computed from an empty room recording (≥ 5 min) measured before starting the MEG experiment (< 1 h) for each participant using an identical MEG machine with the same acquisition setting. Using the forward model and noise covariance matrix, the source signals of MEG data were computed using the weighted minimum norm estimation (wMNE) method (Lin et al., 2006), which restricts the sources of the inverse problem by minimising the energy (L2 norm), while weighting the deep sources to facilitate their detection. This algorithm was selected because (i) it was recommended as a default option in Brainstorm and (ii) it is suitable for cases where a template brain is used instead of individual MR images, which only returns rough approximations of the forward model and is unsuitable for other inversion algorithms (such as beamformer), which require a better model approximation than the wMNE method. The orientation of the neural sources was restricted to be normal to the cortex. The data obtained from the source reconstruction process consisted of epoch time-series signals for each of the 15,000 cortical vertices. High-dimensional data were limited to 68 anatomical regions of interest (ROIs) defined by the Desikan-Killiany atlas (Desikan et al., 2006) by averaging the signals of the vertices included in each anatomical region. During averaging, the signs of the signals were flipped in the vertices, where the normal orientation was opposite to the dominant orientation of the corresponding region. For visual inspection, the ROI time series were averaged across trials under the same conditions (FF, MF, and MB) and categories (biological and non-biological) and across all or occipital ROIs. Finally, to obtain the non-phase-locked induced oscillatory power of the MEG data, a time-frequency (TF) analysis was performed. TF data were computed as envelopes of the epoched ROI time series before averaging, which were extracted using the Hilbert transform. The signals were narrow-band filtered for delta (δ, 2–4 Hz), theta (θ, 5–7 Hz), alpha (α, 8–12 Hz), beta (β, 15–29 Hz), low-gamma (lγ, 30–48 Hz), and high-gamma (hγ, 52–90 Hz) frequency activities, and their envelopes were computed as power of the Hilbert transform for each frequency band. The frequency windows for gamma bands (lγ and hγ) were designed to avoid power line noise at 50 Hz. The envelopes were flattened by multiplying the amplitudes by frequency (1/f compensation), normalised against a baseline period from −1,000 to −500 ms (ITI), and used in the statistical analyses.

### 2.7 Statistical analysis

#### 2.7.1 Behavioural data

Statistical analyses of behavioural data were performed using MATLAB, the Statistics and Machine Learning Toolbox (MathWorks), and the Multiple Testing Toolbox (Martínez-Cagigal, 2021).

First, for each participant, the 20 stimuli with the highest ‘*biologi-ness*’ rating were assigned to the biological category, and the lowest 20 were assigned to the non-biological category. Therefore, different stimuli were included in the biological and non-biological categories for each participant. This categorisation was used for subsequent statistical analyses.

Pre-rating scores (‘*biologi-ness*’, *object saliency*, *facial saliency*, *liking*, and *beauty*) were compared using a non-parametric bootstrapping approach to examine the differences in aesthetic appeal levels between biological and non-biological categories. Participant-wise differences in each rating were computed between categories, which were then averaged using resampling with replacement data across all participants 10,000 times. The percentage of resampled averages larger or smaller than 0 (the smaller value) determined the significance level (*P*-value). Next, non-parametric correlations between the five pre-rating scores were examined within each stimulus category (biological and non-biological) using a bootstrapping approach. For each score pair, participant-wise Spearman’s *rho* was computed, which were averaged using resampling with replacement data across all participants 10,000 times. The percentage of resampled average coefficients larger or smaller than 0 (the smaller value) determined the significance level (*P*-value). In the pre-rating data analysis, bootstrapping analysis was performed repeatedly, which increased the risk of Type I error (Curran-Everett, 2000). Therefore, to control for the false discovery rate (FDR), *P*-values were adjusted using the Benjamini–Hochberg method (Benjamini and Hochberg, 1995). The *P*-values were also multiplied by two to account for the two-tailed test.

Prior to analysing the responses to the CFS task, trials in the FF and MF conditions were further subdivided into two subsets based on the categories of the presented stimuli (biological and non-biological), which resulted in the CFS trials being categorised into five conditions, each containing 20 trials: FF-biological, FF-non-biological, MF-biological, MF-non-biological, and MB. Responses to Q2 (‘beauty?’ question) were compared between the five conditions by calculating the proportions of trials that responded as ‘yes’ to Q2 (proportion of ‘beauty’ response). These comparisons were made using the bootstrapping approach with FDR correction. Finally, to examine whether presentations of aesthetically appealing stimuli modified behavioural responses to Q2 (‘beauty?’ question), pre-rating scores were compared between stimuli presented in ‘beauty’ and ‘non-beauty’ trials within each condition (FF-biological, FF-non-biological, MF-biological, and MF-non-biological). These comparisons were also made using the bootstrapping approach with FDR correction. The grand mean and 95% bootstrap confidence interval of the statistics (i.e., mean proportion) are reported.

#### 2.7.2 MEG data

Statistical analyses of MEG data were performed using MATLAB (MathWorks, Natick, MA, USA), Fieldtrip toolbox (Maris and Oostenveld, 2007; Oostenveld et al., 2011), Statistics and Machine Learning Toolbox (MathWorks, Natick, MA, USA), and Multiple Testing Toolbox (Martínez-Cagigal, 2021). Initially, the temporal resolution of TF data was reduced from 2,000 to 250 Hz to accommodate the large TF data in the permutation tests and achieve realistic computational costs in terms of time and memory. The downsampled TF data in the target time window (0–3,500 ms) were used for statistical analysis.

First, to examine differences in induced brain activity between FF (conscious) and MF (non-conscious) conditions, normalised regional TF data were compared between the conditions for each category (FF-biological vs. MF-biological, FF-non-biological vs. MF-non-biological) using a non-parametric cluster-based permutation approach implemented in the Fieldtrip toolbox (Maris and Oostenveld, 2007). This approach is used broadly for statistically examining TF data under the control of the family-wise error rate (FWER) in clinical (Machetanz et al., 2021; Dell’Acqua et al., 2022, 2023; Hoshi et al., 2024) and basic (Piai et al., 2012; Popov et al., 2018; Stegemöller et al., 2021) research. It involves two major steps: cluster definition and significance calculation. During cluster definition, for each pair of conditions, the *T*-statistic of the dependent samples was calculated in the TF space (time: 876 × frequency: 6) for each region (68) (observed statistics). Notably, these *T*-statistics were only used for cluster definition and not for statistical inference or for calculating the significance probability of the cluster. The *T*-statistic was also computed using a shuffled dataset across participants and conditions 1,000 times, generating a probability distribution of random statistics. For each data point in the TF space, the observed statistics were examined to determine whether they were above or below the critical value (0.025) in the right or left tail of the random probability distribution, respectively. Data points where the observed statistics exceeded the tails were clustered based on their temporal and frequency adjacencies on each side of the tail and defined as positive and negative clusters. This procedure empirically defines ‘clusters,’ encompassing multiple adjacent data points in the TF data which exhibit similar behaviours concerning the effect of interest (*T*-statistics). For significance calculation, the observed *T*-statistic was summed in each cluster (observed cluster statistics), while the random distribution of cluster statistics (i.e., null hypothesis distribution) was generated by collecting the maximum of summed *T*-statistics among detected clusters in the shuffled data for each of the 1,000 iterations (‘maxsum’ method in the cluster-based permutation algorithm in the Fieldtrip toolbox). The proportion of the null hypothesis distribution that was larger (for positive clusters) or smaller (for negative clusters) than the observed cluster statistics was considered as the significance level (*P*-value) of each observed cluster. The *P*-values were multiplied by two to account for the two-tailed test. This approach controlled the FWER at 0.05 because the *P*-value of each cluster was derived from its ranking in the null hypothesis distribution, which was shaped dynamically by the number of comparisons and correlations between data points (Maris and Oostenveld, 2007; Groppe et al., 2011). For each cluster, the size of the cluster, average *T*-statistic across data points included in each cluster, and *P*-value multiplied by two to account for the two-tailed test were reported. Additionally, a peak was defined as the data point in the TF space with the maximum absolute *T*-statistics in each cluster, whose *T*-statistic, time, and frequency were reported.

Next, to investigate the relationship between the aesthetic appeal level of the stimuli and neural responses, correlations between the pre-rating scores (*object saliency*, *facial saliency*, *liking*, and *beauty*) and normalised regional TF data were examined for each condition (FF-biological, FF-non-biological, MF-biological, and MF-non-biological). For each score, non-parametric correlations with the TF data (Spearman’s *rho*) were computed in the TF space (time: 876 × frequency: 6) for each participant and region (68) (correlation map). The statistical significance of the correlation maps was assessed using the non-parametric cluster-based permutation approach. For each correlation map, a one-sample *T*-statistic against zero was obtained in the TF space (time: 876 × frequency: 6) (observed statistics). Cluster definition and significance calculation followed the same procedure for the FF vs. MF comparisons. For each cluster, we reported the cluster size, average *T*-statistic across data points included in each cluster, and *P*-value, as well as the *T*-statistic, time, and frequency of a peak in each cluster. Notably, while the cluster-based permutation approach addressed the multiple comparison issue between TF data points by controlling for the FWER (Maris and Oostenveld, 2007), we did not address the problem between multiple pairs of the cluster-based permutation tests (i.e., repeated use of the tests). This was because the analyses were exploratory, investigating the conditional differences in TF data or correlations between pre-rating scores and TF data. These analyses lacked *a priori* hypotheses and focus on specific conditions or pre-ratings.

Finally, to replicate the results without relying on the ‘*biologi-ness*’ rating, a series of statistical analyses were repeated using all data (FF-all and MF-all conditions) without categorising stimuli into biological and non-biological, as detailed in section C of the Supplementary materials.

## 3 Results

### 3.1 Behavioural data

#### 3.1.1 Pre-rating

**FIGURE 2:**
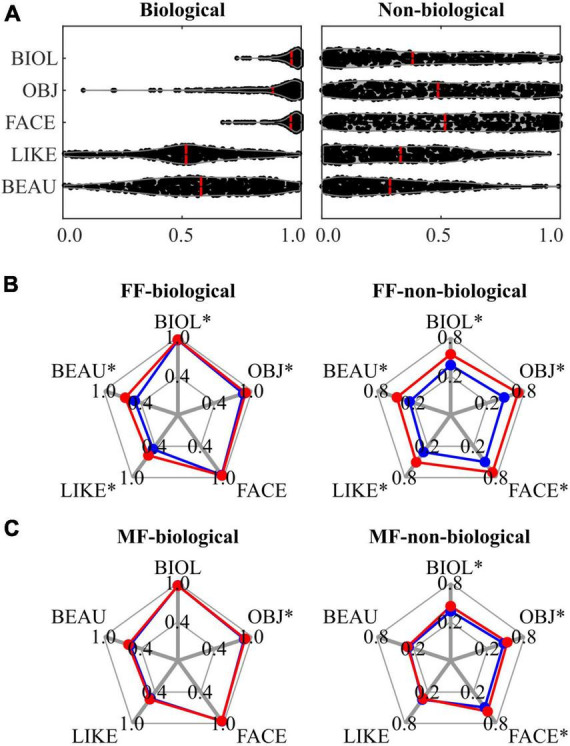
Results of behavioural data analysis for the biological (left panels) and non-biological (right panels) categories. **(A)** A violin plot visualising the distribution of each pre-rating score, with the mean value represented by a red line. **(B)** A spider plot contrasting the mean of each pre-rating score in the FF conditions, in which participants responded as ‘yes’ (red: ‘beauty’) and ‘no’ (blue: ‘non-beauty’) to the Q2 (‘beauty?’ question), respectively. **(C)** A spider plot contrasting the mean of each pre-rating score in the MF conditions, in which participants responded as ‘yes’ (red: ‘beauty’) and ‘no’ (blue: ‘non-beauty’) to the Q2 (‘beauty?’ question), respectively. An asterisk (*) indicates a significant difference in pre-ratings scores between ‘yes’ (‘beauty’ response) and ‘no’ (‘non-beauty’ response) trials. FF, face-face condition; MF, Mondrian-face condition; BIOL, ‘*biologi-ness*’ rating; OBJ, *object saliency* rating; FACE, *facial saliency* rating; LIKE; *liking* rating; BEAU, *beauty* rating.

In this study, the stimuli were pre-rated using five VAS scores (‘*biologi-ness*’, *object saliency*, *facial saliency*, *liking*, and *beauty*), with further subcategorisation into biological and non-biological based on the ‘*biologi-ness*’ score. Supplementary Figure 1 presents the pre-rating results, indicating that stimuli with younger IDs (< #30) received higher ‘*biologi-ness*’, *object saliency*, and *facial saliency* scores. Supplementary Figure 2 summarises the subcategorisation outcomes, revealing that stimuli with IDs < 30 were more frequently allocated to the biological category, whereas those with ID ≥ 30 were allocated to the non-biological category. Figure 2A depicts the raw data for pre-rating scores for each category, demonstrating a ceiling effect in ‘*biologi-ness*’, *object saliency*, and *facial saliency* ratings within the biological category. The biological category scored higher than the non-biological category on all pre-rating scales (all *P* < 0.001, FDR-corrected) (Table 1). Supplementary Figures 3, 4 and Supplementary Tables 3, 4 outline the correlations between scores. In the biological category (Supplementary Figure 3 and Supplementary Table 3), all pairs within the pre-rating scores exhibited positive correlations, except for the *facial saliency* × *beauty* scores. In the non-biological category (Supplementary Figure 4 and Supplementary Table 4), all pairs exhibited positive correlations, except for those that included *liking* scores.

**TABLE 1:** Summary of the pre-rating data.

	Biological category				Non-biological category				
	***M***	***SE***	***CI* (LL)**	***CI* (UL)**	***M***	***SE***	**CI (LL)**	***CI* (UL)**	***P* (FDR)**
BIOL	0.959	0.006	0.946	0.971	0.382	0.040	0.313	0.470	< 0.001*
OBJ	0.881	0.023	0.825	0.917	0.487	0.045	0.409	0.582	< 0.001*
FACE	0.956	0.008	0.936	0.968	0.517	0.039	0.442	0.591	< 0.001*
LIKE	0.517	0.035	0.441	0.578	0.331	0.037	0.257	0.401	< 0.001*
BEAU	0.580	0.037	0.504	0.646	0.286	0.032	0.225	0.346	< 0.001*

An asterisk (*) indicates a significant difference between categories. BIOL, ‘*biologi-ness*’ score; OBJ, *object saliency* score; FACE, *facial saliency* score; LIKE, *liking* score; BEAU, *beauty* score; *M*, mean; *SE*, standard error; *CI* (LL), 95% bootstrap confidence interval (lower limit); *CI* (UL), 95% bootstrap confidence interval (upper limit); *P* (FDR), *P*-values controlled for the false discovery rate.

#### 3.1.2 MEG experiment

During CFS sessions, three presentation conditions were employed: FF (conscious), MF (non-conscious), and MB (Mondrian only). These conditions were further subdivided based on the category of the presented stimuli: FF-biological, FF-non-biological, MF-biological, MF-non-biological, and MB. Binary behavioural responses (‘yes’ or ‘no’) to the question regarding the aesthetic quality of stimuli (Q2; ‘Do you feel beauty from the painting?’) were compared between the conditions. Compared with the MB condition (0.320), the proportion of beauty (‘yes’) responses increased in the FF-biological condition (0.543) (*P* < 0.001), decreased in the FF-non-biological condition (0.115) (*P* < 0.001), and was the same in the MF-biological (0.302) (*P* = 0.573) and MF-non-biological (0.337) (*P* = 0.493) conditions. Comparing between categories, the proportion was higher for the FF-biological condition (0.527) than for the FF-non-biological condition (0.155) (*P* < 0.001), which corresponded to the pre-rating differences (Figure 2A and Table 1). The proportion of beauty responses was not different between the MF-biological and MF-non-biological conditions (*P* = 0.291).

The relationships between the pre-rating and behavioural responses to Q2 are shown in the spider plots in Figures 2B, C and Table 2. For the FF conditions (FF-biological and FF-non-biological) (Figure 2B and Table 2A), all scores were higher for the beauty trials than for the non-beauty trials (red and blue lines in Figure 2B, respectively), except for the *facial saliency* rating in the FF-biological condition. Pre-rating differences were also observed in the MF conditions (MF-biological and MF-non-biological) (Figure 2C and Table 2B) for the *object saliency* rating in the MF-biological condition and ‘*biologi-ness*’, *object saliency*, and *facial saliency* ratings in the MF-non-biological condition.

**TABLE 2:** Summary of the relationships between pre-rating scores and response data.

	Beauty trials				Non-beauty trials				
	***M***	***SE***	***CI* (LL)**	***CI* (UL)**	***M***	***SE***	***CI* (LL)**	***CI* (UL)**	***P* (FDR)**
**(A) FF**									
**Biological**									
%Trials	0.543	0.051			0.457	0.051			
BIOL	0.961	0.006	0.949	0.972	0.956	0.008	0.939	0.969	0.008*
OBJ	0.898	0.020	0.850	0.930	0.859	0.027	0.800	0.904	< 0.001*
FACE	0.959	0.008	0.937	0.971	0.951	0.010	0.922	0.966	0.099
LIKE	0.575	0.033	0.506	0.633	0.455	0.037	0.385	0.523	< 0.001*
BEAU	0.654	0.034	0.580	0.713	0.501	0.037	0.429	0.569	< 0.001*
**Non-biological**									
%Trials	0.115	0.023			0.885	0.023			
BIOL	0.535	0.074	0.394	0.676	0.372	0.051	0.289	0.486	0.009*
OBJ	0.694	0.066	0.550	0.805	0.499	0.055	0.405	0.614	0.004*
FACE	0.698	0.066	0.553	0.809	0.519	0.049	0.422	0.610	0.001*
LIKE	0.506	0.054	0.395	0.601	0.342	0.042	0.254	0.417	< 0.001*
BEAU	0.472	0.052	0.367	0.567	0.279	0.036	0.205	0.343	< 0.001*
**(B) MF**									
**Biological**									
%Trials	0.302	0.048			0.698	0.048			
BIOL	0.958	0.008	0.942	0.972	0.960	0.007	0.946	0.973	0.407
OBJ	0.890	0.025	0.830	0.929	0.865	0.030	0.799	0.915	0.019*
FACE	0.953	0.012	0.923	0.970	0.957	0.008	0.936	0.969	0.368
LIKE	0.537	0.032	0.474	0.594	0.534	0.047	0.444	0.623	0.845
BEAU	0.602	0.036	0.533	0.665	0.569	0.047	0.472	0.652	0.208
**Non-biological**									
%Trials	0.337	0.055			0.663	0.055			
BIOL	0.440	0.042	0.366	0.528	0.339	0.050	0.261	0.454	0.004*
OBJ	0.519	0.048	0.441	0.625	0.438	0.053	0.344	0.547	0.001*
FACE	0.571	0.041	0.489	0.647	0.465	0.048	0.382	0.567	0.001*
LIKE	0.325	0.040	0.246	0.398	0.338	0.049	0.246	0.431	0.633
BEAU	0.300	0.034	0.237	0.364	0.280	0.039	0.208	0.356	0.217
**(C) MB**									
%Trials	0.320	0.050			0.680	0.050			

An asterisk (*) indicates a significant difference between the Beauty vs. Non-beauty trials. FF, face-face condition; MF, Mondrian-face condition; MB, Mondrian-background condition; BIOL, ‘*biologi-ness*’ score; OBJ, *object saliency* score; FACE, *facial saliency* score; LIKE, *liking* score; BEAU, *beauty* score; *M*, mean; *SE*, standard error; *CI* (LL), 95% bootstrap confidence interval (lower limit); *CI* (UL), 95% bootstrap confidence interval (upper limit); *P* (FDR), *P*-values controlled for the false discovery rate.

### 3.2 MEG data

#### 3.2.1 Visual inspection of waveforms

**FIGURE 3:**
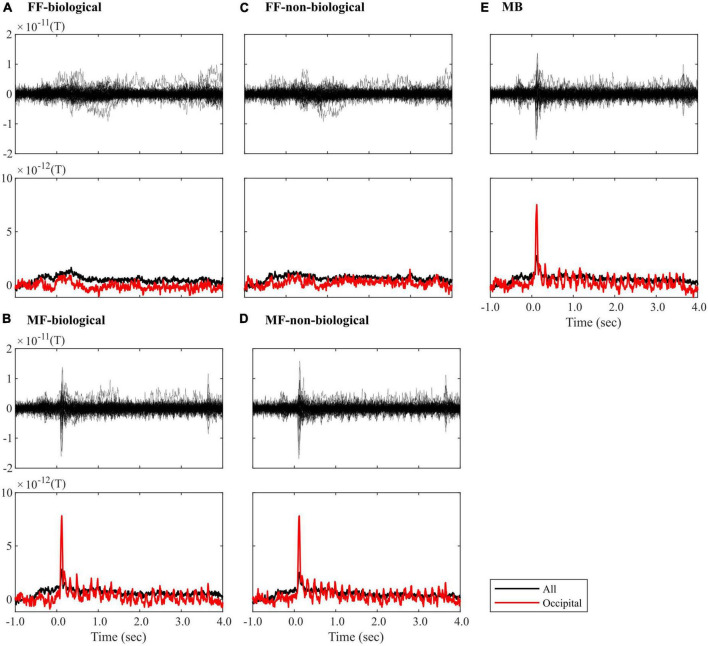
ROI time-series averaged within trials of the **(A)** FF-biological, **(B)** MF-biological, **(C)** FF-non-biological, **(D)** MF-non-biological, and **(E)** MB conditions. The top plot in each panel visualises the butterfly plot, where each line represents each of the 68 ROIs. The bottom plot in each panel visualises the root-mean-square (RMS) waveform, where the signals were averaged across all (black line) or occipital (red line) ROIs. All waveforms were corrected for baseline (–100 to 0 ms). FF, face-face condition; MF, Mondrian-face condition; MB, Mondrian-background condition.

Figure 3 illustrates the average ROI time series signals for each condition (FF-biological, FF-non-biological, MF-biological, MF-non-biological, and MB). Clear visual responses to the flashing Mondrian images (6.25 Hz) were observed under MF and MB conditions (Figures 3B, D, E). A strong event-related field (ERF) appeared at the onset of Mondrian flashes (approximately 100 ms after onset), with amplitudes reducing in the later time windows but repeating periodically until the end of the flashes (3,500 ms). The oscillatory ERF was evident in the occipital ROIs (red lines in Figures 3B, D, E). No explicit ERF was identified in the FF condition, except for weak signal fluctuations between 0 and 1,500 ms (Figures 3A, C). No clear difference was observed between the biological and non-biological categories.

#### 3.2.2 TF data: conscious vs. non-conscious conditions

**FIGURE 4:**
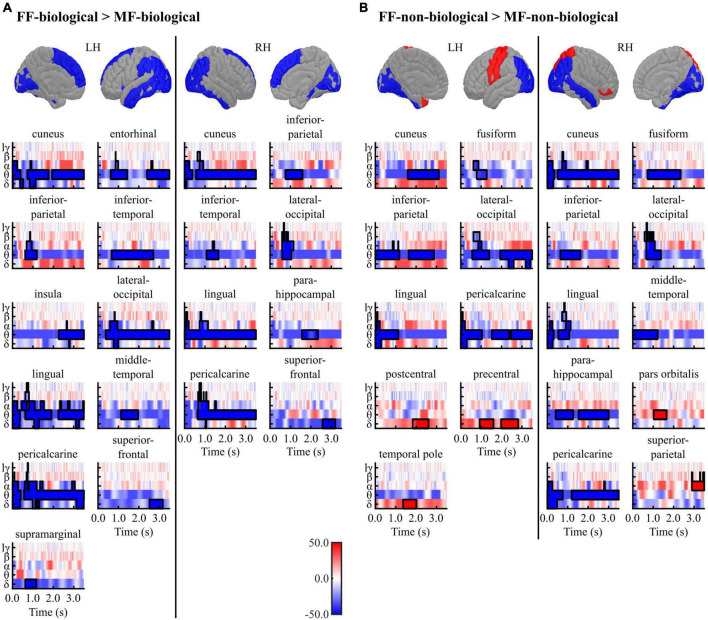
Results of the cluster-based permutation tests between the FF vs. MF conditions for the **(A)** biological and **(B)** non-biological categories. All TF images are scaled equally, with significant clusters emphasised using black bold lines. The regions (ROIs) in the template brain images were coloured red when any positive clusters were found in the ROI or blue when any negative clusters were found in the ROI. The results for hγ band are not displayed, because no significant clusters were found in the band. FF, face-face condition; MF, Mondrian-face condition; LH, left hemisphere; RH, right hemisphere; lγ, low-gamma; hγ, high-gamma.

Figure 4 and Tables 3, 4 summarise the differences observed in induced brain activity (TF data) between the FF (conscious) and MF (non-conscious) conditions for each category (biological and non-biological). Overall, the results revealed strong frequency-tagged responses to Mondrian images in the MF condition, in addition to category-specific responses to facial stimuli, which differed between the biological and non-biological categories. In both stimulus categories, the MF condition induced higher theta and adjacent band activities in the occipitotemporal regions, including the bilateral pericalcarine, lateral occipital, and inferior parietal cortices, lingual gyri, cuneus, and right parahippocampal cortex, than those of the FF condition, which were sustained throughout the trial (0–3,500 ms) (Figure 4 and Tables 3, 4). In the biological category (Figure 4A and Table 3), the FF condition also showed lower frequency activities in the bilateral superior frontal and inferior temporal cortices, and the left middle temporal, entorhinal, and insula cortices than the MF condition. In the non-biological category (Figure 4B and Table 4), the FF condition exhibited higher delta-band activity in the left precentral and postcentral gyri and temporal pole, higher theta activity in the right pars orbitalis, higher alpha-band activity in the right superior parietal lobule, and lower theta-band activity in the bilateral fusiform and right middle temporal gyri than those in the MF condition.

**TABLE 3:** Results of the cluster-based permutation tests for TF data between the FF-biological and MF-biological conditions.

	Cluster			Peak				
**Direction**	**Size**	***T* (mean)**	***P***	***T***	**Time (ms)**	**Frequency**	**LR**	**Region**
Negative	392	−3.095	0.006	−4.505	2,632	Theta	L	Cuneus
	345	−3.444	0.006	−5.094	988	Theta	L	Cuneus
	200	−3.120	0.040	−3.946	156	Alpha	L	Cuneus
	293	−3.131	0.006	−4.554	3,404	Theta	L	Entorhinal
	240	−3.095	0.006	−3.954	1,148	Theta	L	Entorhinal
	214	−2.587	0.024	−3.315	696	Theta	L	Inferior parietal
	512	−2.877	0.002	−3.958	1,960	Theta	L	Inferior temporal
	327	−2.943	0.008	−3.795	3,440	Theta	L	Insula
	950	−3.268	0.002	−4.330	828	Theta	L	Lateral occipital
	623	−2.838	0.002	−4.201	908	Alpha	L	Lingual
	412	−2.853	0.008	−4.443	2,668	Theta	L	Lingual
	242	−3.366	0.024	−4.291	100	Theta	L	Lingual
	210	−2.593	0.044	−3.187	1,460	Theta	L	Middle temporal
	1,121	−2.895	0.002	−4.877	896	Theta	L	Pericalcarine
	267	−3.070	0.026	−4.224	92	Alpha	L	Pericalcarine
	163	−2.833	0.028	−3.450	2,864	Delta	L	Superior frontal
	137	−3.115	0.040	−3.795	928	Delta	L	Supramarginal
	815	−3.378	0.002	−5.970	2,780	Theta	R	Cuneus
	178	−3.230	0.032	−4.034	124	Theta	R	Cuneus
	210	−2.549	0.028	−3.475	1,220	Theta	R	Inferior parietal
	165	−2.996	0.030	−4.867	1,296	Theta	R	Inferior temporal
	310	−3.031	0.008	−5.283	884	Alpha	R	Lateral occipital
	1,046	−3.298	0.004	−4.593	72	Theta	R	Lingual
	198	−2.363	0.032	−2.688	1,832	Theta	R	Parahippocampal
	910	−2.836	0.002	−4.805	1,072	Alpha	R	Pericalcarine
	157	−2.838	0.042	−3.734	2,756	Delta	R	Superior frontal

*T* (mean), *T*-statistics averaged across data points included in each cluster; *P*, *P*-values; *T*, *T*-statistics.

**TABLE 4:** Results of the cluster-based permutation tests for TF data between the FF-non-biological and MF-non-biological conditions.

	Cluster			Peak				
**Direction**	**Size**	***T* (mean)**	***P***	***T***	**Time (ms)**	**Frequency**	**LR**	**Region**
Positive	195	2.639	0.034	3.259	2,164	Delta	L	Postcentral
	170	3.629	0.018	4.309	1,380	Delta	L	Precentral
	211	2.519	0.022	3.150	2,156	Delta	L	Precentral
	163	2.429	0.048	2.839	1,840	Delta	L	Temporal pole
	158	2.624	0.040	3.399	1,488	Theta	R	Pars orbitalis
	181	2.842	0.036	3.985	3,340	Alpha	R	Superior parietal
Negative	383	−2.896	0.008	−4.059	2,116	Theta	L	Cuneus
	186	−2.408	0.042	−3.112	784	Alpha	L	Fusiform
	312	−2.719	0.008	−3.845	100	Theta	L	Inferior parietal
	313	−2.569	0.008	−3.556	2,280	Theta	L	Inferior parietal
	511	−3.286	0.006	−4.517	3,408	Theta	L	Lateral occipital
	331	−3.475	0.008	−6.802	796	Beta	L	Lateral occipital
	403	−2.972	0.002	−4.965	904	Theta	L	Lingual
	385	−2.675	0.006	−3.726	276	Theta	L	Pericalcarine
	260	−3.028	0.008	−4.046	2,712	Theta	L	Pericalcarine
	214	−2.587	0.024	−2.979	2,108	Theta	L	Pericalcarine
	831	−3.025	0.010	−4.264	2,572	Theta	R	Cuneus
	226	−2.686	0.036	−4.191	60	Alpha	R	Cuneus
	406	−2.497	0.002	−2.973	1,548	Theta	R	Fusiform
	247	−2.937	0.006	−4.414	856	Theta	R	Inferior parietal
	364	−3.031	0.004	−4.784	932	Alpha	R	Lateral occipital
	256	−3.025	0.020	−5.671	752	Beta	R	Lingual
	177	−3.036	0.036	−3.763	60	Theta	R	Lingual
	305	−2.735	0.014	−3.677	100	Theta	R	Middle temporal
	370	−2.665	0.010	−3.283	1,720	Theta	R	Parahippocampal
	216	−2.835	0.040	−3.524	764	Theta	R	Parahippocampal
	590	−2.434	0.008	−3.136	1,476	Theta	R	Pericalcarine
	368	−2.844	0.016	−3.899	136	Alpha	R	Pericalcarine

*T* (mean), *T*-statistics averaged across data points included in each cluster; *P*, *P*-values; *T*, *T*-statistics.

#### 3.2.3 TF data: correlations to pre-ratings in the biological category

**FIGURE 5:**
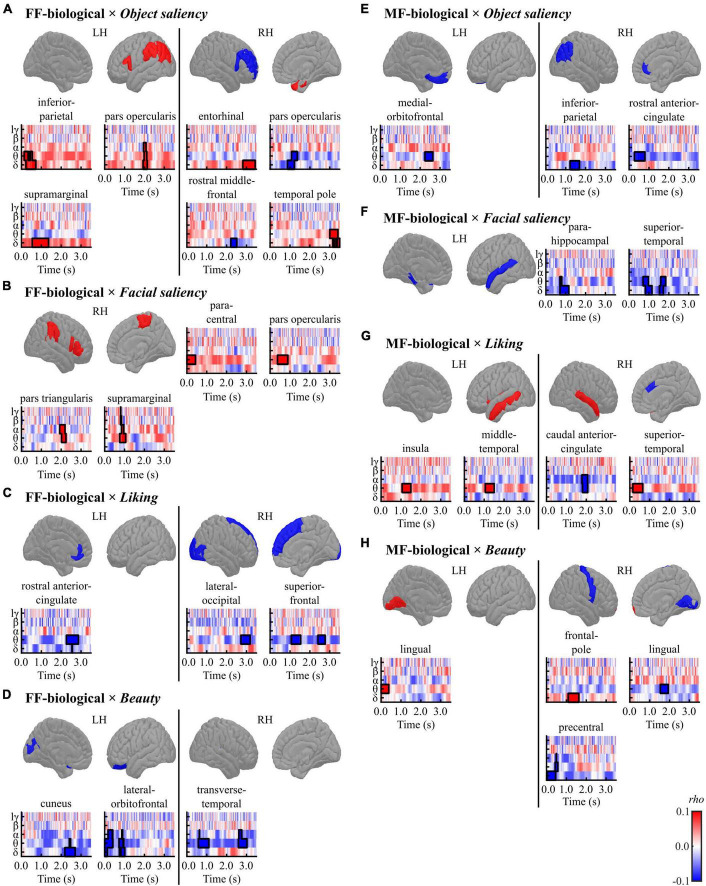
Results of the cluster-based permutation tests for evaluating correlations between regional TF data and pre-rating scores [**(A,E)** *object saliency*, **(B,F)** *facial saliency*, **(C,G)** *liking*, and **(D,H)** *beauty*] in each condition [**(A–D)** FF-biological and **(E–H)** MF-biological] in the biological category. All TF images are scaled equally, with significant clusters emphasised using black bold lines. The regions (ROIs) in the template brain images were coloured red when any positive clusters were found in the ROI or blue when any negative clusters were found in the ROI. The results for hγ band are not displayed, because no significant clusters were found in the band. FF, face-face condition; MF, Mondrian-face condition; LH, left hemisphere; RH, right hemisphere; lγ, low-gamma; hγ, high-gamma.

The correlations between induced brain activities (TF data) and four pre-rating scores (*object saliency*, *facial saliency*, *liking*, and *beauty*) were examined within each condition (FF-biological, MF-biological, FF-non-biological, and MF-non-biological). Figure 5 and Table 5 present a summary of the results for the conditions in the biological category (FF-biological and MF-biological).

**TABLE 5:** Results of the cluster-based permutation tests for evaluating correlations between the TF data and pre-rating scores for the biological category.

	Cluster			Peak				
**Direction**	**Size**	***T* (mean)**	***P***	***T***	**Time (ms)**	**Frequency**	**LR**	**Region**
**(A) FF-biological × *Object saliency***								
Positive	205	2.323	0.030	2.989	652	Delta	L	Inferior parietal
	121	2.623	0.046	3.669	2,024	Alpha	L	Pars opercularis
	206	3.313	0.002	4.798	1,028	Delta	L	Supramarginal
	155	3.278	0.004	4.999	2,968	Delta	R	Entorhinal
	173	2.500	0.014	3.571	3,096	Theta	R	Temporal pole
Negative	158	−2.782	0.016	−4.211	1,104	Delta	R	Pars opercularis
	76	−3.924	0.040	−5.571	2,392	Delta	R	Rostral middle frontal
**(B) FF-biological × *Facial saliency***								
Positive	109	3.323	0.030	4.203	276	Theta	R	Paracentral
	130	2.886	0.012	3.655	576	Theta	R	Pars opercularis
	124	2.843	0.020	3.651	2,000	Alpha	R	Pars triangularis
	122	2.788	0.032	3.745	904	Alpha	R	Supramarginal
**(C) FF-biological × *Liking***								
Negative	153	−2.925	0.020	−4.139	2,384	Theta	L	Rostral anterior cingulate
	117	−3.397	0.032	−5.505	3,020	Theta	R	Lateral occipital
	119	−3.227	0.014	−4.536	1,420	Theta	R	Superior frontal
	91	−3.072	0.048	−4.092	2,564	Theta	R	Superior frontal
**(D) FF-biological × *Beauty***								
Negative	147	−3.137	0.008	−4.759	2,472	Delta	L	Cuneus
	195	−3.064	0.002	−4.738	0	Delta	L	Lateral orbitofrontal
	121	−2.600	0.050	−3.563	928	Delta	L	Lateral orbitofrontal
	145	−2.849	0.026	−3.427	2,852	Theta	R	Transverse temporal
	134	−2.749	0.034	−3.634	996	Theta	R	Transverse temporal
**(E) MF-biological × *Object saliency***								
Negative	101	−3.090	0.044	−4.034	2,412	Theta	L	Medial orbitofrontal
	119	−3.055	0.024	−4.258	1,440	Delta	R	Inferior parietal
	132	−2.531	0.036	−2.945	512	Theta	R	Rostral anterior cingulate
**(F) MF-biological × *Facial saliency***								
Negative	133	−2.948	0.018	−4.003	936	Delta	L	Parahippocampal
	152	−2.683	0.024	−3.238	1,052	Delta	L	Superior temporal
	113	−2.755	0.038	−3.869	1,716	Theta	L	Superior temporal
**(G) MF-biological × *Liking***								
Positive	110	3.589	0.030	4.494	1,356	Theta	L	Insula
	112	3.930	0.010	5.948	1,360	Theta	L	Middle temporal
	125	3.521	0.012	4.789	416	Theta	R	Superior temporal
Negative	137	−2.613	0.020	−3.499	2,012	Alpha	R	Caudal anterior cingulate
**(H) MF-biological × *Beauty***								
Positive	97	3.236	0.038	4.341	252	Theta	L	Lingual
	134	2.843	0.032	3.901	1,560	Delta	R	Frontal pole
Negative	100	−3.045	0.042	−3.956	1,848	Theta	R	Lingual
	145	−2.532	0.030	−3.034	472	Theta	R	Precentral

FF, face-face condition; MF, Mondrian-face condition; *T* (mean), *T*-statistics averaged across data points included in each cluster; *P*, *P*-values; *T*, *T*-statistics.

In the FF (conscious)-biological condition, the *object saliency* score was positively correlated with low-frequency activities in the left inferior parietal cortex, left pars opercularis, left supramarginal gyrus, right entorhinal cortex, and right temporal pole and negatively correlated with delta-band activity in the right pars opercularis and rostral middle frontal gyrus (Figure 5A and Table 5A). The *facial saliency* score was positively correlated with low-frequency activities in the right paracentral and supramarginal gyri, pars opercularis, and pars triangularis (Figure 5B and Table 5B). The *liking* score was negatively correlated with low-frequency activities in the left rostral anterior cingulate cortex (rACC), right lateral occipital cortex and right superior frontal gyrus (Figure 5C and Table 5C). The *beauty* score was negatively correlated with low-frequency activities in the left cuneus, left lateral orbitofrontal cortex (lOFC), and right transverse temporal gyrus (Figure 5D and Table 5D).

In the MF (non-conscious)-biological condition, the *object saliency* score was negatively correlated with low-frequency activities in the left medial orbitofrontal cortex (mOFC), right inferior parietal lobule, and right rACC (Figure 5E and Table 5E). The *facial saliency* score was negatively correlated with low-frequency activities in the left parahippocampal and superior temporal cortices (Figure 5F and Table 5F). The *liking* score was positively correlated with theta-band activity in the left insula, left middle temporal cortex, and right superior temporal cortex and negatively correlated with alpha-band activity in the right caudal ACC (cACC) (Figure 5G and Table 5G). Finally, the *beauty* score was positively correlated with low-frequency activities in the left lingual gyrus and right frontal pole and negatively correlated with theta-band activity in the right lingual cortex and precentral gyrus (Figure 5H and Table 5H).

#### 3.2.4 TF data: correlations to pre-ratings in the non-biological category

**FIGURE 6:**
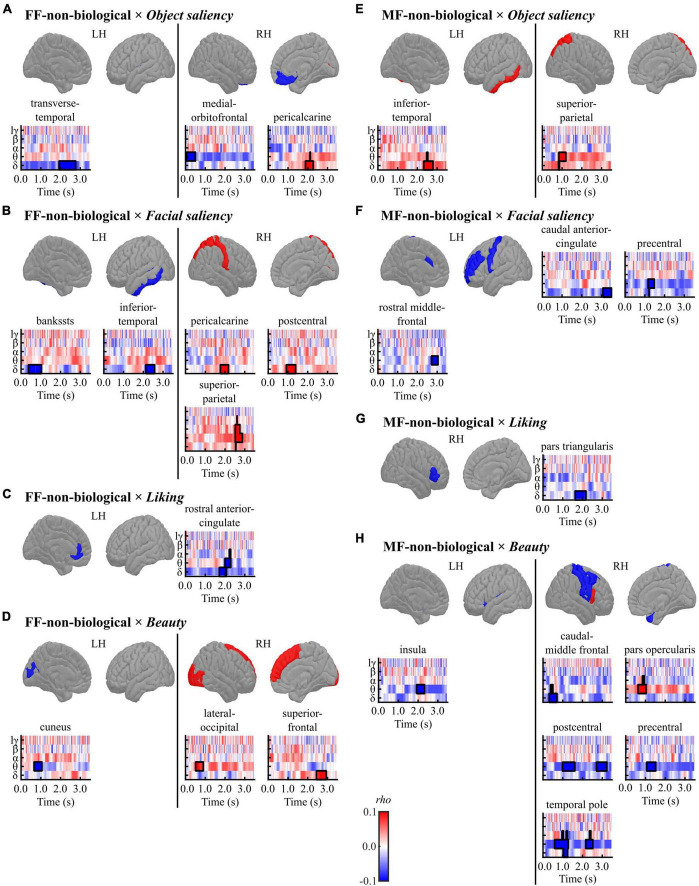
Results of the cluster-based permutation tests for evaluating correlations between regional TF data and pre-rating scores [**(A,E)** *object saliency*, **(B,F)** *facial saliency*, **(C,G)** *liking*, and **(D,H)** *beauty*] in each condition [**(A–D)** FF-non-biological and **(E–H)** MF-non-biological] in the non-biological category. All TF images are scaled equally, with significant clusters emphasised using black bold lines. The regions (ROIs) in the template brain images were coloured red when any positive clusters were found in the ROI or blue when any negative clusters were found in the ROI. The results for hγ band are not displayed, because no significant clusters were found in the band. FF, face-face condition; MF, Mondrian-face condition; LH, left hemisphere; RH, right hemisphere; lγ, low-gamma; hγ, high-gamma.

Figure 6 and Table 6 present a summary of the results for the conditions in the non-biological category (FF-non-biological and MF-non-biological).

**TABLE 6:** Results of the cluster-based permutation tests for evaluating correlations between the TF data and pre-rating scores for the non-biological category.

	Cluster			Peak				
**Direction**	**Size**	***T* (mean)**	***P***	***T***	**Time (ms)**	**Frequency**	**LR**	**Region**
**(A) FF-non-biological × *Object saliency***								
Positive	105	3.598	0.020	5.493	2,020	Delta	R	Pericalcarine
Negative	211	−2.672	0.012	−3.398	2,152	Delta	L	Transverse temporal
	110	−3.451	0.044	−4.323	264	Theta	R	Medial orbitofrontal
**(B) FF-non-biological × *Facial saliency***								
Positive	103	3.291	0.044	4.987	2,080	Delta	R	Pericalcarine
	117	2.709	0.038	3.428	1,236	Delta	R	Postcentral
	153	2.501	0.036	3.336	2,720	Alpha	R	Superior parietal
Negative	165	−2.765	0.022	−3.463	568	Delta	L	Bankssts
	111	−3.791	0.010	−5.329	2,324	Delta	L	Inferior temporal
**(C) FF-non-biological × *Liking***								
Negative	175	−3.126	0.008	−4.059	2,184	Theta	L	Rostral anterior cingulate
**(D) FF-non-biological × *Beauty***								
Positive	99	3.642	0.034	6.273	656	Theta	R	Lateral occipital
	124	2.523	0.042	2.977	2,540	Delta	R	Superior frontal
Negative	99	−3.144	0.050	−4.108	860	Theta	L	Cuneus
**(E) MF-non-biological × *Object saliency***								
Positive	133	2.909	0.028	4.071	2,624	Delta	L	Inferior temporal
	102	2.963	0.048	3.985	1,040	Theta	R	Superior parietal
**(F) MF-non-biological × *Facial saliency***								
Negative	109	−4.353	0.006	−5.652	3,372	Delta	L	Caudal anterior cingulate
	81	−4.097	0.024	−6.420	1,328	Theta	L	Precentral
	81	−3.802	0.050	−4.954	2,892	Theta	L	Rostral middle frontal
**(G) MF-non-biological × *Liking***								
Negative	136	−2.797	0.026	−3.376	1,684	Delta	R	Pars triangularis
**(H) MF-non-biological × *Beauty***								
Positive	125	3.144	0.038	4.746	908	Theta	R	Pars opercularis
Negative	101	−3.499	0.030	−5.229	2,080	Theta	L	Insula
	121	−2.592	0.032	−3.329	624	Delta	R	Caudal middle frontal
	156	−3.907	0.008	−5.839	1,256	Theta	R	Postcentral
	132	−2.864	0.030	−3.497	3,176	Theta	R	Postcentral
	114	−2.973	0.038	−3.427	1,320	Theta	R	Precentral
	255	−2.910	0.002	−3.990	1,096	Delta	R	Temporal pole
	108	−2.817	0.046	−3.974	2,416	Theta	R	Temporal pole

FF, face-face condition; MF, Mondrian-face condition; *T* (mean), *T*-statistics averaged across data points included in each cluster; *P*, *P*-values; *T*, *T*-statistics.

In the FF (conscious)-non-biological condition, the *object saliency* score was positively correlated with delta-band activity in the right pericalcarine cortex and negatively correlated with low-frequency activities in the left transverse temporal gyrus and right mOFC (Figure 6A and Table 6A). The *facial saliency* score was positively correlated with low-frequency activities in the right pericalcarine and superior parietal cortices and right postcentral gyrus and negatively correlated with delta-band activity in the left bankssts (cortical areas around the superior temporal sulcus [STS]) and left inferior temporal cortex (Figure 6B and Table 6B). The *liking* score was negatively correlated with theta-band activity in the left rACC (Figure 6C and Table 6C). The *beauty* score was positively correlated with low-frequency activities in the right lateral occipital cortex and superior frontal gyrus and negatively correlated with theta-band activity in the left cuneus (Figure 6D and Table 6D).

In the MF (non-conscious)-non-biological condition, the *object saliency* score was positively correlated with low-frequency activities in the left inferior temporal cortex and the right superior parietal lobule (Figure 6E and Table 6E). The *facial saliency* score was negatively correlated with low-frequency activities in the left cACC, left precentral gyrus, and left rostral middle frontal gyrus (Figure 6F and Table 6F). The *liking* score was negatively correlated with delta-band activity in the right pars triangularis (Figure 6G and Table 6G). Finally, the *beauty* score was positively correlated with theta-band activity in the right pars opercularis and negatively correlated with low-frequency activities in distributed regions, including the left insula, right temporal pole, right caudal middle frontal, postcentral, and precentral gyri (Figure 6H and Table 6H).

## 4 Discussion

This study revealed two main findings: (i) the non-conscious presentation of portrait paintings induced spatiotemporally distributed low-frequency brain activities for both the biological and non-biological categories, and (ii) these brain activities exhibited distinct patterns between the biological and non-biological categories and between the conscious and non-conscious conditions.

In this study, we examined the induced MEG signals recorded during a CFS task, where aesthetically appealing facial stimuli, subcategorised into biological and non-biological stimuli, were non-consciously presented to participants. We hypothesised that the non-conscious presentation of aesthetically appealing stimuli would induce neural responses related to emotional processing if aesthetic emotion holds evolutionary significance for humans. For the biological and non-biological categories, aesthetic appeal induced MEG responses, even when suppressed using CFS (MF conditions) (Figures 5E–H, 6E–H, and Tables 5E–H, 6E–H), thereby supporting our hypothesis. However, the neural responses to the aesthetic appeal of stimuli differed between (i) biological vs. non-biological categories and (ii) conscious vs. non-conscious presentations in the spatial, temporal, and oscillatory senses. In the following sections, we highlight the variations (i) in behavioural data (section 4.1) and discuss the interaction between (i) and (ii) in MEG data (sections 4.2, 4.3 and 4.4), where the results in the FF (conscious)-biological (section 4.2), MF (non-conscious)-biological (section 4.3), and non-biological (FF and MF) (section 4.4) conditions were introduced.

### 4.1 Behavioural-level differences in aesthetic processes of biological and non-biological stimuli

In this study, we subcategorised the stimuli into two subsets based on the ‘*biologi-ness*’ score of the pre-ratings by each participant. Despite controlling for low-level features, qualitative differences between the categories influenced the results. Overall, the biological category scored higher on all pre-ratings than the non-biological category (Table 1), supporting the processing fluency theory of aesthetic pleasure (Reber et al., 1998, 2004). This theory proposes that ‘the more fluently perceivers can process an object, the more positive their aesthetic response,’ where prototypicality is a variable influencing fluency. In the present study, the biological category scored higher in the ‘*biologi-ness*’ rating, where the faces were depicted more clearly (higher in *object* and *facial saliency* ratings). Hence, as these stimuli were more prototypical than the other facial stimuli, they were processed more fluently, leading to enhanced affective ratings, such as *liking* and *beauty*, as exemplified by positive correlations between the rating scores (Supplementary Table 3). However, in the non-biological category, the correlation revealed that the *liking* score was independent of ‘*biologi-ness*’, *object saliency*, and

*facial saliency* scores (Supplementary Table 4). This indicated that factors beyond processing fluency influenced the aesthetic *liking* scores because the non-biological stimuli were low in prototypicality and their fluent processes were interrupted (Reber et al., 1998, 2004). The pleasure-interest model of aesthetic liking (PIA model) (Graf and Landwehr, 2015) distinguishes aesthetic

liking based on its construction mechanisms. Pleasure-based liking is influenced by processing fluency and triggered by bottom-up stimulus-driven automatic processing, whereas interest-based liking is elicited by the reduction of disfluency during top-down perceiver-driven controlled processing (Graf and Landwehr, 2017). For non-biological stimuli, including paintings in abstract styles (such as cubism, abstract art, and surrealism), participants are more likely to engage in deeper active elaboration to resolve the disfluency of the abstract paintings, triggering interest-based aesthetic liking. Therefore, interest-based liking is assumed to be another contributor to *liking* scores for non-biological stimuli. Additionally, the integration of processing fluency and learning theories for making future predictions provides explanations of aesthetic processing (Brielmann and Dayan, 2022; Starr, 2023). Within this line of research, the role of predictive processing (PP) has recently been receiving attention as a mental function accounting for the aesthetic experience (Frascaroli et al., 2024). This framework suggests that encountering unpredictable stimuli (e.g., artworks), which deviates from the schema of the viewers, gives rise to prediction error and ‘uncertainty,’ whose resolution (i.e., change) drives a positive affective experience (Van De Cruys et al., 2024). When presenting the stream of portrait paintings in our study, participants may have naturally expected the presence of facial images, and the presentation of less typical facial stimuli, namely the ones in the non-biological category, may have increased prediction error and uncertainty. Contemplation or elaborations of such stimuli might have led to the resolution of uncertainty (e.g., identification of facial images or finding them to ‘make sense’), which consequently enhanced the aesthetic pleasure measured by the *liking* score. In addition to interest-based liking, this serves as a unique source of the *liking* score in non-biological stimuli. Notably, the PP framework complicates the processing fluency theory that hedonic experience driven by fluency is biassed by prior expectation to the stimuli or fluency itself (e.g., ‘more fluent than expected,’ ‘prefer to be kept in a state of puzzlement’) (Yoo et al., 2024). Furthermore, the prototypicality of the stimuli, which is a key driver of the aesthetic emotion in processing fluency theory, is formed by repeated PP throughout the life of the human being. As exemplified by the ‘other-race effect’ (Pesciarelli et al., 2021; Stelter and Schweinberger, 2023) and facial familiarity effects (Axelrod et al., 2015), the ‘biological’ visual features for carrying evolutionary important information vary individually, although they share some common characteristics, such as symmetry and good proportion. This indicates that the ‘personally’ formed prototypical representation of an object, which is updated daily through individual active inferences (i.e., PP), is considered to carry evolutionarily important information and has prioritised access to the emotional system. Therefore, the PP framework not only plays a role as a unique contributor to aesthetic emotion for non-biological stimuli but also serves as a generative function of prototypical images and influences fluency-driven aesthetic emotion. Upon examining the association between pre-ratings and behavioural responses during the CFS task (Figures 2B, C and Table 2), we found that all pre-rating scores, except the *facial saliency* score, differed between beauty and non-beauty responses in the FF (conscious)-biological condition (Figure 2B and Table 2A). Moreover, a weak link between the *facial saliency* and *beauty* ratings was demonstrated by the low correlation coefficients (Supplementary Table 3). This finding may be attributed to a ceiling effect on the *facial saliency* score in biological stimuli (Figure 2A). Owing to the strong correlation between *facial saliency* and ‘*biologi-ness*’ scores and selection criteria that only retained stimuli with very high ‘*biologi-ness*’ scores in the biological category, both scores hit the ceiling. Consequently, correlations between *facial saliency* and ‘*biologi-ness*’ scores with other pre-rating scores were lower for the biological category than those of the non-biological category, which did not experience this effect. Notably, the non-consciously presented stimuli modified the behavioural responses during the CFS task, such that the *object saliency* score was higher for beauty than for non-beauty responses in the MF-biological condition, while the ‘*biologi-ness*’ and *object* and *facial saliency* scores were higher for beauty than for non-beauty responses in the MF-non-biological condition (Table 2B). This implies that non-conscious neural inputs of salient stimuli may bias subsequent aesthetic labelling behaviour. Previous studies have revealed that non-consciously presented faces undergo not only emotional, but also perceptual and cognitive processes (Axelrod et al., 2015), such as discrimination of face vs. scrambled face (Jiang and He, 2006), upright vs. inverted faces (Jiang et al., 2007; Stein et al., 2012), and familiarity (Gobbini et al., 2013). As image saliency directly influences perceptual and cognitive processes, it may interfere with the perceptual and/or cognitive fluency of non-conscious processing of facial stimuli and change their gut-level feelings captured by guessing the aesthetic quality of covert stimuli. This aligns with a concept of ‘unfelt’ fluency, which occurs on a lower, perceptual, and sub-personal level, reflects the level of matches between perceptual information with basic visual expectations derived from our visual systems (i.e., prototypical patterns of neural activity, determined congenitally and formed by PP), and operates as a non-conscious process (Brouillet and Friston, 2023). As *beauty* and *liking* scores are the results of conscious aesthetic evaluations, they are dynamic reflections of various perceptual and cognitive processes, such as fluency, elaboration, and prediction. However, in the non-conscious presentation scenario, the top-down regulative process is inactive for stimuli with weak intensity (such as subliminal stimuli) (Baars, 1988; Dehaene et al., 1998), indicating that only limited perceptual and cognitive processes can contribute to aesthetic emotion. For example, the interest-based liking system and PP for resolving uncertainty require a top-down regulation of incoming information to drive aesthetic emotion; thus, these would not be involved in the processing of non-consciously presented stimuli. Therefore, in the present study, the pleasure-based liking system, driven by ‘unfelt’ processing fluency, was likely dominant for aesthetic processing in the MF condition. The behavioural responses to the non-consciously presented stimuli were modified by the *saliency* scores but not by the *beauty* or *liking* scores, indicating that non-consciously presented information accesses limited processes associated with the perceptual saliency of the stimuli, such as the ‘unfelt’ fluency; hence, the behavioural response observed was likely based upon such limited information. Therefore, the results of the aesthetic labelling based on limited information (behavioural responses to the non-consciously presented stimuli) would be distinctive from those based on comprehensive information (*beauty* and *liking* scores in the pre-rating).

### 4.2 Conscious aesthetic processing of biological stimuli

After highlighting the behavioural differences between the biological and non-biological categories in section 4.1 we discussed the interaction between biological vs. non-biological and conscious vs. non-conscious contrasts in the correlation analysis between the pre-ratings and TF data. Here, we have focused on the results in the most representative FF (conscious)-biological condition (Figures 5A–D and Tables 5A–D) and structured the following paragraphs to sequentially discuss spatial, oscillatory, and temporal dimensions for clarity.

Regarding the spatial (regional) dimension of the results, the *saliency* scores were positively correlated with low-frequency activities (from the delta to alpha bands) in parietal and temporal regions (Figures 5A, B). These results aligned with those of previous studies indicating that emotional faces induce delta and theta band activities, particularly in occipitotemporal regions (Güntekin and Başar, 2014). Moreover, the level of processing fluency in facial stimuli was associated with neural responses in occipitotemporal and parietal regions (Natu and O’Toole, 2011). Multiple face-selective areas in the occipital and temporal cortices include the occipital face area (Haxby et al., 1999; Gauthier et al., 2000), fusiform face area (Kanwisher et al., 1997), posterior part of the superior temporal sulcus (Kanwisher et al., 1997; Hoffman and Haxby, 2000), anterior temporal lobe (Tsao et al., 2008; Rajimehr et al., 2009), and anterior superior temporal sulcus (Pitcher et al., 2011), which border the clusters identified relevant to the *saliency* scores. Stimuli with higher *saliency* scores were processed more fluently and exhibited enhanced slow band activities in these regions, signifying involvement of bottom-up perceptual and cognitive processes related to visual images. In contrast, affective processing of the stimuli, quantified by *liking* and *beauty* scores, negatively correlated with low-frequency activities mainly in frontal regions, such as the lOFC and superior frontal gyrus (Figures 5C, D). Previous studies have demonstrated that prefrontal regions respond to aesthetic ratings of various stimuli (Nakamura et al., 1998; Kawabata and Zeki, 2004; Di Dio et al., 2007; Winston et al., 2007; Kirk, 2008; Chatterjee et al., 2009; Ishizu and Zeki, 2011; Kedia et al., 2014; Martín-Loeches et al., 2014; Ferrari et al., 2015, 2017; Chuan-Peng et al., 2020). This indicates that reductions in low-frequency activities in the ‘emotional brain’ coincide with the arousal of aesthetic emotions.

Upon exploring the oscillatory characteristics of the induced response in the FF-biological condition, perceptual/cognitive (*object* and *facial saliency*) and affective (*liking* and *beauty*) processes exhibited correlations in the same low-frequency activities, but in different regions and opposite directions. While the former exhibited positive relationships (Figures 5A, B), the latter exhibited negative relationships (Figures 5C, D). Similar contradictory results have been reported in studies on emotional faces, with some reporting augmented or induced event-related synchronisation (ERS) in delta and theta band activities (Aftanas et al., 2001, 2004; Balconi and Pozzoli, 2007, 2009; Başar et al., 2008; Balconi et al., 2009b,a; Bamidis et al., 2009; Güntekin and Başar, 2009, 2014; Knyazev et al., 2009), while others reporting their attenuation or event-related desynchronisation (ERD) (Balconi and Lucchiari, 2006; Balconi and Pozzoli, 2009). This inconsistency between ERS and ERD during emotional picture processing has also been documented in alpha and lower-beta bands (Schubring and Schupp, 2021). These discrepancies may be explained by the physiological antagonism between cognitive and affective processes in fluency-driven aesthetic processing (Hoshi and Menninghaus, 2018). A previous study demonstrated that highly affective texts predict larger pupil dilations, whereas highly fluent texts predict smaller pupil dilations (Hoshi and Menninghaus, 2018), indicating that the cognitive and affective processes of aesthetically appealing stimuli cause antagonistic reactions to the autonomic nervous system. Briefly, the balance between sympathetic and parasympathetic reactions drives pupillary dilation, with excitement in sympathetic activity and inhibition of parasympathetic activity evoking dilation, and vice versa (van der Wel and van Steenbergen, 2018). Although studies focusing on the relationship between task-related autonomic nervous system reactions and cortical oscillatory activity are limited, one study revealed that autonomic behaviour is associated with delta and theta band responses to emotional stimuli (Balconi et al., 2015). These findings imply that fluency-driven aesthetic processes induce cognitive and affective responses in antagonistic directions in delta and theta band activities in the central nervous system. Previous studies on visual emotional processing have revealed inconsistent results between ERS and ERD using standard picture groups, such as ‘Pictures of Facial Affect’ (Ekman and Friesen, 1976) and ‘International Affective Picture System’ (Lang et al., 1997). Researchers typically assumed that these standardised sets would be equally processed by all individuals in both cognitive and affective senses. However, the potential variabilities in cognitive and affective processes were often overlooked in these studies, which may have led some studies to emphasise cognitive processing and others to emphasise affective processing induced by the stimulus set, resulting in contradictory findings.

Upon examining the temporal dynamics in the FF-biological condition, we found that neural processes occur in multiple time windows. Remarkably, for the *beauty* score, a negative cluster was identified in a very early time frame in the left lOFC (preparation and fading-in phases, 0–1000 ms) (Figure 5D). Previous neuroaesthetics studies have used electrophysiological measurements, such as electroencephalography (EEG) (Jacobsen and Höfel, 2003; Muñoz and Martín-Loeches, 2015; Sarasso et al., 2020) and MEG (Cela-Conde et al., 2004, 2013), to study the temporal dimensions of aesthetic processing. Among these, two studies have suggested that aesthetic processing comprises two temporally distinct steps. One EEG-based study (Jacobsen and Höfel, 2003) examined event-related potential and found that graphical patterns judged as ‘not beautiful’ evoked early frontal negativity after 300 ms, whereas judgements of their symmetry evoked sustained posterior negativity approximately 600 ms after stimulus onset. Another MEG-based study (Cela-Conde et al., 2013) using paintings as stimuli reported distinct connectivity patterns between two time windows: an early window (250–750 ms), characterised by dense local connections within the occipital region and extending links to orbitofrontal regions for making quick judgements regarding beauty, and the later window (1,000–1,500 ms), with distributed global connection, which was active only for ‘beautiful’ stimuli and involved in appraisals of detailed aspects of beauty. Despite their different timeframes, these two studies reported temporally distinct processes: early processes related to aesthetic emotion for making quick impressions and later processes for detailed evaluations of stimuli. This two-step process corresponds to the temporal dynamics observed in our study, where early low-frequency activities in the OFC were associated with the affective dimension of aesthetic appeal. As the OFC is a core region for the subjective experience of aesthetic emotion, namely beauty (Ishizu and Zeki, 2011), the early processes might be related to aesthetic emotion for making quick impressions. Subsequently, low-frequency activities were induced by cognitive and affective processes in the other brain regions, where the stimuli could be aesthetically evaluated in detail.

### 4.3 Non-conscious aesthetic processing of biological stimuli

In contrast to the FF (conscious)-biological condition discussed in section 4.2 we focused on the results of the correlation analysis in the MF (non-conscious)-biological condition (Figures 5E–H and Tables 5E–H).

Before discussing the results in the non-conscious condition, it is worth noting that the differences between conscious and non-conscious conditions were strongly biassed by frequency-tagged responses (steady-state visually evoked field) (Parkkonen et al., 2008; Vialatte et al., 2010) to Mondrian images, which entail robust visual evoked responses at a frequency corresponding to the refreshing rate of the presented stimuli. To optimise suppression effects (Zhu et al., 2016; Drewes et al., 2018), we set the refresh rate of the Mondrian images to 6.25 Hz (section 2.4). Visual inspection of the ROI time series (Figure 3) revealed clear ERFs at approximately 6 Hz under MF and MB conditions. The results of the TF data should be interpreted with a consideration of frequency-tagged responses in the theta band (5–7 Hz). As Mondrian images were presented throughout the time window of interest (0–3,500 ms), the sustained activity increased in the theta band, which was commonly found for both categories (biological and non-biological), should be considered as frequency-tagged responses to the Mondrian images. Sustained augmentation of theta band activity was found in the bilateral pericalcarine, lateral occipital, and inferior parietal cortices, lingual gyri, cuneus, and right parahippocampal cortex in the MF conditions (MF-biological and MF-non-biological) (Figure 4). The stimulus-related activities found in these regions must be contaminated by frequency-tagged responses and should be interpreted with caution.

In this section, the results of the MF-biological condition are discussed for *saliency* scores (*object* and *facial saliency* scores) and affective scores (*liking* and *beauty*). *Saliency* scores were negatively associated with low-frequency activity in the fronto-temporal regions (Figures 5E, F), such as the mOFC, rACC, parahippocampal, superior temporal, and inferior parietal cortices. The temporal regions overlapped with the face-selective areas, such as the anterior and posterior parts of the superior temporal sulcus (Duchaine and Yovel, 2015), indicating the involvement of bottom-up visual processing related to the facial images. However, the correlation direction was negative, which is opposite from that of temporal and parietal clusters identified in the FF-biological condition (Figures 5A, B). Additionally, more negative clusters were exclusively found in the frontal and limbic regions in the MF-biological condition, such as the mOFC and rACC (Figure 5E). Despite being designed to capture perceptual and cognitive processing of the stimuli, the *saliency* score was sensitive to affective processing (Supplementary Table 3). Furthermore, as demonstrated by the behavioural data (section 4.1), the pleasure-based liking system, driven by ‘unfelt’ processing fluency, was considered dominant for aesthetic processing in the MF condition. As *saliency* scores were directly linked to perceptual fluency, the association between the *saliency* score and affective processing may have been more emphasised in the MF-biological condition than in the FF-biological condition, leading the distinct correlation patterns between the two conditions. The negative correlations in the frontal and limbic regions found for *saliency* scores in the MF-biological condition (Figure 5E) were similar to the results for affective ratings in the FF-biological condition (Figures 5C, D). For example, the ACC, a part of the reward circuit (Haber and Knutson, 2009), is responsive to aesthetic emotions (Kawabata and Zeki, 2004; Vartanian and Goel, 2004; Brown et al., 2011; Tsukiura and Cabeza, 2011; Boccia et al., 2016), and is often coactivated with the OFC. Taken together, we can infer that the *saliency* scores likely became more strongly associated with affective processing during aesthetic labelling of non-consciously presented images, which resulted in negative clusters in the frontal and limbic regions and modifications of behavioural responses.

Regarding the affective ratings (*liking* and *beauty*) in the MF-biological condition, the results showed two types of correlations: (i) negative correlations in low-frequency activities in the cACC and precentral and right lingual gyri, and (ii) positive correlations in low-frequency activities in the temporal region, left lingual gyrus, and frontal pole (Figures 5G, H). For the negative correlations, the cluster in ACC may capture the affective processing of the covert stimuli, akin to the overt stimuli (Figure 5C). Additionally, the negative cluster in the precentral gyrus may also be related to the affective response as the presentation of non-beautiful, ‘ugly,’ or aversive stimuli is associated with neural responses in the precentral and postcentral gyri (Kawabata and Zeki, 2004; Ishizu and Zeki, 2011; Hayes and Northoff, 2012). Traditionally, it is assumed that the non-conscious processing of emotional information follows a rapid subcortical pathway, bypassing the slower cortical route to directly relay information to the amygdala and ‘emotion system’ (LeDoux, 1996; Tamietto and De Gelder, 2010). Therefore, it would be reasonable that the non-consciously presented biological stimuli with lower *beauty* scores induced fast and low-frequency activities in the precentral gyrus during the very early time window, approximately 500 ms after stimulus onset. For the positive correlations, clusters were primarily found in the occipitotemporal regions at the theta band. Although the significant clusters were temporally limited, the TF maps (Figures 5G, H) revealed sustained theta band activities throughout the stimulus presentation period. This requires cautious interpretation, given that the pattern indicates frequency-tagged responses to the Mondrian images used in the CFS paradigm. While the exact reason for the correlations between affective ratings and frequency-tagged responses remains unknown, we can infer that the properties of the masked stimuli can influence the masking effect of the CFS, including the paradigm known as ‘breaking CFS’ (Jiang et al., 2007; Stein et al., 2011). Thus, the affective properties of the biological stimuli, which are associated with low-frequency brain activities (Figures 5C, D), may modulate the frequency-tagged responses induced by CFS in the similar frequency band (theta).

### 4.4 Conscious and non-conscious aesthetic processing of non-biological stimuli

The results for non-biological stimuli were different from those for biological stimuli. In the FF (conscious)-non-biological condition, the *object saliency* score was negatively correlated with the low-frequency activities in the mOFC (Figures 6A, B), suggesting associations between perceptual/cognitive saliency and affective process. As discussed in section 4.1 fluency, as well as the interest-based liking system and PP framework, contribute to driving the aesthetic emotion aroused by non-biological stimuli. Our results revealed traces of these processes in non-biological stimuli. For example, the bankssts (STS), where the negative correlation was found with the *facial saliency* score (Figure 6B), is involved in detecting social information in various stimuli, such as biological motion and eye gaze, and relaying the information to other regions for further processing (Iidaka, 2014; Deen et al., 2015). Similarly, another study revealed that the STS innervates the amygdala to deliver emotional information about facial stimuli (Pitcher et al., 2017). This indicates that the bankssts were involved in the interest-based liking system and PP, contributing to resolving disfluency or uncertainty in stimuli by detecting social and emotional cues in the abstract, non-biological images and relaying the information to the other regions to modify emotional responses. Additionally, there is substantial evidence that awareness of ambiguous perceptual images modulates the activities in V1 (Tong, 2003; Zeki, 2004). The positive correlations between *saliency* scores and low-frequency activity in the pericalcarine cortex (V1) (Figures 6A, B) may reflect the relationship between perceptual *saliency* and awareness (i.e., resolving disfluent and uncertain states). Similarly, the superior parietal cortex, where the *facial saliency* score was positively correlated to the theta/alpha-band activities, has been associated with the disambiguation process of bistable images (Kanai et al., 2011). Furthermore, the inferior temporal cortex is famously associated with object recognition (Logothetis and Sheinberg, 1996; Conway, 2018); thus, the negative cluster identified in this region for *facial saliency* score would indicate the contribution of the recognition process to disambiguate the visual information to drive affective processing. Although the neurological mechanisms underlying the interest-based liking system and PP framework in the context of aesthetic emotion remain unknown, these regions may play a pivotal role in the processes. Regarding the affective scores, the *liking* score correlated negatively with the low-frequency activity in the rACC (Figure 6C), consistent with biological stimuli (Figure 5C), supporting the link between ACC activity and aesthetic pleasure. The *beauty* score correlated with theta-band activity in the occipital regions, such as the lateral occipital cortex and cuneus (Figure 6D), reflecting that the level of aesthetic emotion was associated with the visual information processing, such as the fluency, elaboration, and prediction. Notably, the role of cortical areas in such visual information processing cannot be specified because we did not measure detailed behavioural information for speculating the dominant mental processes underlying aesthetic emotion, which may have varied stimulus-wise (e.g., processing fluency, interest-based liking, or PP). Therefore, our interpretations of the MEG data do not exclude other possibilities; for example, the involvement of the inferior temporal cortex may not reflect its contribution to the disambiguation process (related top-down interest-based liking system and PP framework), but rather the level of prototypicality of the stimuli (related to bottom-up fluency processes).

In the MF (non-conscious)-non-biological condition, *facial saliency* score was negatively correlated with low-frequency activity in the frontal and limbic regions, including the cACC, precentral, and rostral middle frontal gyri (Figure 6F), similar to negative clusters observed in the frontal and limbic regions for biological stimuli (MF-biological condition) (Figure 5E). As discussed in section 4.3 the negative correlations likely indicate the increased link between *saliency* scores and affective processing during the aesthetic labelling of non-consciously presented images. This suggests that, for non-consciously presented stimuli, regardless of their categories, the pleasure-based liking system driven by ‘unfelt’ processing fluency was likely dominant for aesthetic processing, and the other ‘top-down’ frameworks were largely suppressed. The *liking* and *beauty* scores in the MF-non-biological condition demonstrated negative low-frequency clusters, mainly in the fronto-temporal and central regions (Figures 6G, H). Negative clusters in the central region were also found for the biological category under the same condition (Figure 5H), which would be indicative of aversive responses to the ‘non-beautiful’ stimuli. The neural processing for the non-consciously presented aversive stimuli may share similar neural systems across categories. Negative clusters were also found in the caudal middle frontal gyrus and insular cortex (Figure 6H). The dorsolateral prefrontal cortex, including the middle frontal gyrus, has been shown to be responsive to aesthetic emotion (Cela-Conde et al., 2004; Cattaneo et al., 2014). Similarly, the insula is considered to be a region responsive to emotional (Singer et al., 2009; Gasquoine, 2014; Uddin et al., 2017) and aesthetically appealing stimuli (Di Dio et al., 2007; Di Dio and Vittorio, 2009; Brown et al., 2011; Chatterjee and Vartanian, 2014; Boccia et al., 2016). These findings indicate that the non-conscious presentation of non-biological stimuli induced neural responses related to aesthetic emotion, observed as negative correlations in the low-frequency bands.

### 4.5 Limitations

This study has four main limitations. First, the CFS method for non-conscious presentation imposed technical constraints; the stimuli faded in and out to enhance the suppression effects, allowing us to study only the induced components (oscillations) of the MEG signals, which were contaminated by the signals induced by the Mondrian mask at 6.25 Hz. Future studies investigating this topic should consider using time-locked evoked activities. Second, owing to environmental constraints, the number of stimuli (trials) and pre-rating scales were limited. The MEG system was installed in a hospital setting and regularly used for clinical examinations; thus, the experimental procedures were designed to be completed within a limited time slot, restricting the number of trials in the CFS task, variations in stimuli, and pre-ratings. Although the stimuli were carefully selected (section 2.2), they did not cover before the Renaissance periods or modern art styles. In addition, owing to the limited number of pre-rating scales, the results may be limited to specific positively valenced aesthetic emotions (liking and beauty), preventing generalisability to other negatively valenced aesthetic emotions (Istók et al., 2009; Augustin et al., 2012; Knoop et al., 2016). Third, the ratings and neural responses were obtained non-simultaneously and separated by a gap of up to 5 days between the pre-rating and MEG experiment, because of the aforementioned environmental factors and to reduce the physical burden among participants (section 2.3). Repeated exposure is known to alter the hedonic experience to the stimuli (Montoya et al., 2017). Moreover, prior exposure to the stimuli (i.e., learning) might reduce the experiences of unconformity and uncertainty to the unexpected (i.e., non-biological) stimuli in a PP sense, which may only trigger a ‘faint copy’ of uncertainty resolution (Van De Cruys et al., 2024). As such, the actual in-the-moment aesthetic experience may not be captured, but the stimuli would be labelled as aesthetically appealing owing to their past affordance of the aesthetic experience. Therefore, the aesthetic processes operated during the pre-rating and MEG measurements might not be quantitatively and qualitatively identical. Thus, our findings should be replicated in a future study using a simultaneous recording design. Fourth, the definitions of biological and non-biological stimuli were arbitrary. We operationally defined the biological and non-biological categories as extremes of the single continuous scale of ‘*biologi-ness*’ (section 1), but this idea needs further experimental support. Additionally, the stimuli sets were selected based on the relative scores within each participant (the top and bottom 20 images in the ‘*biologi-ness*’ rating). Another categorisation approach, such as the use of the absolute ‘*biologi-ness*’ score, may show different results; however, this is beyond the scope of the present study and should be investigated further in future psychological and neuroscientific studies.

## 5 Conclusion

This study examined the interaction between consciousness and aesthetic emotions using an electrophysiological method. The results revealed that the non-conscious presentation of biological and non-biological stimuli induced low-frequency brain activities associated with aesthetic ratings, suggesting that aesthetic emotions have adaptive significance. The underlying neural processes were distinctive for each stimulus category, possibly because of different aesthetic processing mechanisms, such as processing fluency, active elaboration, and PP. Furthermore, we discovered that the induced activities differed between the conscious and non-conscious conditions, with the latter thought to emphasise fluency-driven affective processing and suppress top-down regulative processes. Neural responses to aesthetic processing are determined by the interplay between perceptual, cognitive, and affective processes, which are further modified by the intervention of consciousness. This study provides the first empirical evidence supporting the evolutionary significance of aesthetic processing, motivating future studies to clarify the complex relationships among aesthetic processing, facial processing, and consciousness.

## Data availability statement

The datasets presented in this study can be found in online repositories. The names of the repository/repositories and accession number(s) can be found below: Hoshi, H. (2023). MEGstudy on aesthetic emotion. *Mendeley Data*. 1. doi: 10.17632/8v8p82cy23.1.

## Ethics statement

The studies involving humans were approved by the Ethics Committee of Hokuto Hospital and Osaka Metropolitan University Graduate School of Medicine. The studies were conducted in accordance with the local legislation and institutional requirements. The participants provided their written informed consent to participate in this study.

## Author contributions

HH: Conceptualization, Data curation, Formal Analysis, Funding acquisition, Methodology, Project administration, Resources, Software, Visualization, Writing – original draft, Writing – review & editing. AI: Conceptualization, Supervision, Writing – review & editing, Methodology. YS: Data curation, Supervision, Writing – review & editing, Conceptualization. TY: Conceptualization, Funding acquisition, Project administration, Supervision, Writing – review & editing.

## Footnotes

1
https://www.wikiart.org/

2
http://neuroimage.usc.edu/brainstorm

3
http://surfer.nmr.mgh.harvard.edu/

## References

[B1] Aftanas L. I. Reva N. V. Varlamov A. A. Pavlov S. V. Makhnev V. P. (2004). Analysis of evoked EEG synchronization and desynchronization in conditions of emotional activation in humans: Temporal and topographic characteristics. *Neurosci. Behav. Physiol.* 34 859–867. 10.1023/B:NEAB.0000038139.39812.EB/METRICS 15587817

[B2] Aftanas L. I. Varlamov A. A. Pavlov S. V. Makhnev V. P. Reva N. V. (2001). Affective picture processing: Event-related synchronization within individually defined human theta band is modulated by valence dimension. *Neurosci. Lett.* 303 115–118. 10.1016/S0304-3940(01)01703-7 11311506

[B3] Alpers G. W. Gerdes A. B. M. Lagarie B. Tabbert K. Vaitl D. Stark R. (2009). Attention and amygdala activity: An fMRI study with spider pictures in spider phobia. *J. Neural Transm.* 116 747–757. 10.1007/S00702-008-0106-8/FIGURES/4 18726545

[B4] Ames D. L. Fiske S. T. (2010). Cultural neuroscience. *Asian J. Soc. Psychol.* 13 72–82. 10.1111/J.1467-839X.2010.01301.X 23874143 PMC3714113

[B5] Augustin M. D. Wagemans J. Carbon C. C. (2012). All is beautiful? Generality vs. specificity of word usage in visual aesthetics. *Acta Psychol.* 139 187–201. 10.1016/J.ACTPSY.2011.10.004 22123506

[B6] Axelrod V. Bar M. Rees G. (2015). Exploring the unconscious using faces. *Trends Cogn. Sci.* 19 35–45. 10.1016/J.TICS.2014.11.003 25481216

[B7] Baars B. (1988). *A Cognitive Theory of Consciousness.* Cambridge: Cambridge University Press.

[B8] Baillet S. (2017). Magnetoencephalography for brain electrophysiology and imaging. *Nat. Neurosci.* 20 327–339. 10.1038/nn.4504 28230841

[B9] Balconi M. Brambilla E. Falbo L. (2009a). BIS/BAS, cortical oscillations and coherence in response to emotional cues. *Brain Res. Bull.* 80 151–157. 10.1016/J.BRAINRESBULL.2009.07.001 19591907

[B10] Balconi M. Falbo L. Brambilla E. (2009b). BIS/BAS responses to emotional cues: Self report, autonomic measure and alpha band modulation. *Pers. Individ. Differ.* 47 858–863. 10.1016/J.PAID.2009.07.004

[B11] Balconi M. Grippa E. Vanutelli M. E. (2015). What hemodynamic (fNIRS), electrophysiological (EEG) and autonomic integrated measures can tell us about emotional processing. *Brain Cogn.* 95 67–76. 10.1016/J.BANDC.2015.02.001 25721430

[B12] Balconi M. Lucchiari C. (2006). EEG correlates (event-related desynchronization) of emotional face elaboration: A temporal analysis. *Neurosci. Lett.* 392 118–123. 10.1016/J.NEULET.2005.09.004 16202519

[B13] Balconi M. Pozzoli U. (2007). Event-related oscillations (EROs) and event-related potentials (ERPs) comparison in facial expression recognition. *J. Neuropsychol.* 1 283–294. 10.1348/174866407X184789 19331021

[B14] Balconi M. Pozzoli U. (2009). Arousal effect on emotional face comprehension: Frequency band changes in different time intervals. *Physiol. Behav.* 97 455–462. 10.1016/J.PHYSBEH.2009.03.023 19341748

[B15] Bamidis P. D. Klados M. A. Frantzidis C. Vivas A. B. Papadelis C. Lithari C. (2009). A framework combining delta Event-Related Oscillations (EROs) and synchronisation effects (ERD/ERS) to study emotional processing. *Comput. Intell. Neurosci.* 2009:549419. 10.1155/2009/549419 19609455 PMC2709724

[B16] Başar E. Schmiedt-Fehr C. Öniz A. Başar-Eroğlu C. (2008). Brain oscillations evoked by the face of a loved person. *Brain Res.* 1214 105–115. 10.1016/J.BRAINRES.2008.03.042 18471805

[B17] Benjamini Y. Hochberg Y. (1995). Controlling the false discovery rate: A practical and powerful approach to multiple testing. *J. R. Stat. Soc. Ser. B Methodol.* 57 289–300. 10.1111/j.2517-6161.1995.tb02031.x

[B18] Bignardi G. Ishizu T. Zeki S. (2021). The differential power of extraneous influences to modify aesthetic judgments of biological and artifactual stimuli. *PsyCh. J.* 10 190–199. 10.1002/PCHJ.415 33295099

[B19] Boccia M. Barbetti S. Piccardi L. Guariglia C. Ferlazzo F. Giannini A. M. (2016). Where does brain neural activation in aesthetic responses to visual art occur? Meta-analytic evidence from neuroimaging studies. *Neurosci. Biobehav. Rev.* 60 65–71. 10.1016/J.NEUBIOREV.2015.09.009 26619805

[B20] Bornstein M. H. Ferdinandsen K. Gross C. G. (1981). Perception of symmetry in infancy. *Dev. Psychol.* 17 82–86. 10.1037/0012-1649.17.1.82

[B21] Brielmann A. A. Dayan P. (2022). A computational model of aesthetic value. *Psychol. Rev.* 129 1319–1337. 10.1037/REV0000337 35786988

[B22] Brooks S. J. Savov V. Allzén E. Benedict C. Fredriksson R. Schiöth H. B. (2012). Exposure to subliminal arousing stimuli induces robust activation in the amygdala, hippocampus, anterior cingulate, insular cortex and primary visual cortex: A systematic meta-analysis of fMRI studies. *Neuroimage* 59 2962–2973. 10.1016/J.NEUROIMAGE.2011.09.077 22001789

[B23] Brouillet D. Friston K. (2023). Relative fluency (unfelt vs felt) in active inference. *Conscious. Cogn.* 115:103579. 10.1016/J.CONCOG.2023.103579 37776599

[B24] Brown S. Gao X. Tisdelle L. Eickhoff S. B. Liotti M. (2011). Naturalizing aesthetics: Brain areas for aesthetic appraisal across sensory modalities. *Neuroimage* 58 250–258. 10.1016/J.NEUROIMAGE.2011.06.012 21699987 PMC8005853

[B25] Cai C. Kang H. Kirsch H. E. Mizuiri D. Chen J. Bhutada A. (2019). Comparison of DSSP and tSSS algorithms for removing artifacts from vagus nerve stimulators in magnetoencephalography data. *J. Neural Eng.* 16:066045. 10.1088/1741-2552/ab4065 31476752

[B26] Carlsson K. Petersson K. M. Lundqvist D. Karlsson A. Ingvar M. Öhman A. (2004). Fear and the amygdala: Manipulation of awareness generates differential cerebral responses to phobic and fear-relevant (but nonfeared) stimuli. *Emotion* 4 340–353. 10.1037/1528-3542.4.4.340 15571433

[B27] Carroll J. (1998). Steven Pinker’s cheesecake for the mind. *Philos. Lit.* 22 478–485. 10.1353/PHL.1998.0036 34409987

[B28] Cattaneo Z. Lega C. Flexas A. Nadal M. Munar E. Cela-conde C. J. (2014). The world can look better: Enhancing beauty experience with brain stimulation. *Soc. Cogn. Affect. Neurosci.* 9 1713–1721. 10.1093/SCAN/NST165 24132459 PMC4221210

[B29] Cela-Conde C. J. Garcfa-Prieto J. Ramasco J. J. Mirasso C. R. Bajo R. Munara E. (2013). Dynamics of brain networks in the aesthetic appreciation. *Proc. Natl. Acad. Sci. U. S. A.* 110 10454–10461. 10.1073/PNAS.1302855110/SUPPL_FILE/PNAS.201302855SI.PDF 23754437 PMC3690613

[B30] Cela-Conde C. J. Marty G. Maestú F. Ortiz T. Munar E. Fernández A. (2004). Activation of the prefrontal cortex in the human visual aesthetic perception. *Proc. Natl. Acad. Sci. U. S. A.* 101 6321–6325. 10.1073/PNAS.0401427101 15079079 PMC395967

[B31] Chatterjee A. (2014). *The Aesthetic Brain: How we Evolved to Desire Beauty and Enjoy Art.* New York, NY: Oxford University Press.

[B32] Chatterjee A. Thomas A. Smith S. E. Aguirre G. K. (2009). The neural response to facial attractiveness. *Neuropsychology* 23 135–143. 10.1037/A0014430 19254086

[B33] Chatterjee A. Vartanian O. (2014). Neuroaesthetics. *Trends Cogn. Sci.* 18 370–375. 10.1016/J.TICS.2014.03.003 24768244

[B34] Chuan-Peng H. Huang Y. Eickhoff S. B. Peng K. Sui J. (2020). Seeking the ‘Beauty Center’ in the brain: A meta-analysis of fMRI studies of beautiful human faces and visual art. *Cogn. Affect. Behav. Neurosci.* 20 1200–1215. 10.3758/S13415-020-00827-Z 33089442 PMC8058033

[B35] Conway B. R. (2018). The organization and operation of inferior temporal cortex. *Annu. Rev. Vis. Sci.* 4 381–402. 10.1146/ANNUREV-VISION-091517-034202 30059648 PMC6404234

[B36] Curran-Everett D. (2000). Multiple comparisons: Philosophies and illustrations. *Am. J. Physiol. Regul. Integr. Comp. Physiol.* 279 R1–R8. 10.1152/ajpregu.2000.279.1.r1 10896857

[B37] Dahlén A. D. Schofield A. Schiöth H. B. Brooks S. J. (2022). Subliminal emotional faces elicit predominantly right-lateralized amygdala activation: A systematic meta-analysis of fMRI studies. *Front. Neurosci.* 16:868366. 10.3389/FNINS.2022.868366/BIBTEX PMC9339677 35924231

[B38] Darwin C. (1871). *The Descent of Man, and Selection in Relation to Sex.* London: John Murray.

[B39] Deen B. Koldewyn K. Kanwisher N. Saxe R. (2015). Functional organization of social perception and cognition in the superior temporal sulcus. *Cereb. Cortex* 25 4596–4609. 10.1093/CERCOR/BHV111 26048954 PMC4816802

[B40] Dehaene S. Kerszberg M. Changeux J. P. (1998). A neuronal model of a global workspace in effortful cognitive tasks. *Proc. Natl. Acad. Sci. U. S. A.* 95 14529–14534. 10.1073/PNAS.95.24.14529 9826734 PMC24407

[B41] Dell’Acqua C. Dal Bò E. Moretta T. Palomba D. Messerotti Benvenuti S. (2022). EEG time–frequency analysis reveals blunted tendency to approach and increased processing of unpleasant stimuli in dysphoria. *Sci. Rep.* 12 1–13. 10.1038/s41598-022-12263-9 35581359 PMC9113991

[B42] Dell’Acqua C. Hajcak G. Amir N. Santopetro N. J. Brush C. J. Meyer A. (2023). Error-related brain activity: A time-domain and time-frequency investigation in pediatric obsessive–compulsive disorder. *Psychophysiology* 60:e14216. 10.1111/PSYP.14216 36332634

[B43] d’Errico F. Stringer C. B. (2011). Evolution, revolution or saltation scenario for the emergence of modern cultures? *Philos. Trans. R. Soc. B Biol. Sci.* 366:1060. 10.1098/RSTB.2010.0340 21357228 PMC3049097

[B44] Desikan R. S. Ségonne F. Fischl B. Quinn B. T. Dickerson B. C. Blacker D. (2006). An automated labeling system for subdividing the human cerebral cortex on MRI scans into gyral based regions of interest. *Neuroimage* 31 968–980. 10.1016/j.neuroimage.2006.01.021 16530430

[B45] Di Dio C. Vittorio G. (2009). Neuroaesthetics: A review. *Curr. Opin. Neurobiol.* 19 682–687. 10.1016/J.CONB.2009.09.001 19828312

[B46] Di Dio D. C. Macaluso E. Rizzolatti G. (2007). The golden beauty: Brain response to classical and renaissance sculptures. *PLoS One* 2:e1201. 10.1371/JOURNAL.PONE.0001201 18030335 PMC2065898

[B47] Drewes J. Zhu W. Melcher D. (2018). The edge of awareness: Mask spatial density, but not color, determines optimal temporal frequency for continuous flash suppression. *J. Vis.* 18:12. 10.1167/18.1.12 29362805

[B48] Duchaine B. Yovel G. (2015). A revised neural framework for face processing. *Annu. Rev. Vis. Sci.* 1 393–416. 10.1146/ANNUREV-VISION-082114-035518 28532371

[B49] Ekman P. Friesen W. V. (1976). *Pictures of Facial Affect.* Palo Alto, CA: Consulting Psychologist Press.

[B50] Etcoff N. L. (1999). *Survival of the Prettiest: The Science of Beauty.* New York, NY: Anchor Books.

[B51] Fang Z. Li H. Chen G. Yang J. J. (2016). Unconscious processing of negative animals and objects: Role of the amygdala revealed by fMRI. *Front. Hum. Neurosci.* 10:146. 10.3389/FNHUM.2016.00146/ABSTRACT 27092067 PMC4820445

[B52] Ferrari C. Lega C. Tamietto M. Nadal M. Cattaneo Z. (2015). I find you more attractive …after (prefrontal cortex) stimulation. *Neuropsychologia* 72 87–93. 10.1016/J.NEUROPSYCHOLOGIA.2015.04.024 25912761

[B53] Ferrari C. Nadal M. Schiavi S. Vecchi T. Cela-Conde C. J. Cattaneo Z. (2017). The dorsomedial prefrontal cortex mediates the interaction between moral and aesthetic valuation: A TMS study on the beauty-is-good stereotype. *Soc. Cogn. Affect. Neurosci.* 12 707–717. 10.1093/SCAN/NSX002 28158864 PMC5460046

[B54] Fischl B. (2012). FreeSurfer. *Neuroimage* 62 774–781. 10.1016/J.NEUROIMAGE.2012.01.021 22248573 PMC3685476

[B55] Fonov V. Evans A. McKinstry R. Almli C. Collins D. (2009). Unbiased nonlinear average age-appropriate brain templates from birth to adulthood. *Neuroimage* 47:S102. 10.1016/s1053-8119(09)70884-5

[B56] Frascaroli J. Leder H. Brattico E. Van De Cruys S. (2024). Aesthetics and predictive processing: Grounds and prospects of a fruitful encounter. *Philos. Trans. R. Soc. B.* 379:20220410. 10.1098/RSTB.2022.0410 38104599 PMC10725766

[B57] Gasquoine P. G. (2014). Contributions of the insula to cognition and emotion. *Neuropsychol. Rev.* 24 77–87. 10.1007/S11065-014-9246-9/TABLES/1 24442602

[B58] Gauthier I. Tarr M. J. Moylan J. Skudlarski P. Gore J. C. Anderson A. W. (2000). The fusiform ‘face area’ is part of a network that processes faces at the individual level. *J. Cogn. Neurosci.* 12 495–504. 10.1162/089892900562165 10931774

[B59] Gobbini M. I. Gors J. D. Halchenko Y. O. Rogers C. Guntupalli J. S. Hughes H. (2013). Prioritized detection of personally familiar faces. *PLoS One* 8:e66620. 10.1371/JOURNAL.PONE.0066620 23805248 PMC3689778

[B60] Graf L. K. M. Landwehr J. R. (2015). A dual-process perspective on fluency-based aesthetics: The pleasure-interest model of aesthetic liking. *Personal. Soc. Psychol. Rev.* 19 395–410. 10.1177/1088868315574978 25742990

[B61] Graf L. K. M. Landwehr J. R. (2017). Aesthetic pleasure versus aesthetic interest: The two routes to aesthetic liking. *Front. Psychol.* 8:217446. 10.3389/FPSYG.2017.00015/BIBTEX PMC5276863 28194119

[B62] Grammer K. Fink B. Møller A. P. Thornhill R. (2003). Darwinian aesthetics: Sexual selection and the biology of beauty. *Biol. Rev.* 78 385–407. 10.1017/S1464793102006085 14558590

[B63] Groppe D. M. Urbach T. P. Kutas M. (2011). Mass univariate analysis of event-related brain potentials/fields I: A critical tutorial review. *Psychophysiology* 48 1711–1725. 10.1111/J.1469-8986.2011.01273.X 21895683 PMC4060794

[B64] Güntekin B. Başar E. (2009). Facial affect manifested by multiple oscillations. *Int. J. Psychophysiol.* 71 31–36. 10.1016/J.IJPSYCHO.2008.07.019 18725252

[B65] Güntekin B. Başar E. (2014). A review of brain oscillations in perception of faces and emotional pictures. *Neuropsychologia* 58 33–51. 10.1016/J.NEUROPSYCHOLOGIA.2014.03.014 24709570

[B66] Haber S. N. Knutson B. (2009). The reward circuit: Linking primate anatomy and human imaging. *Neuropsychopharmacol* 35 4–26. 10.1038/npp.2009.129 19812543 PMC3055449

[B67] Hari R. Puce A. (2017). *MEG–EEG Primer.* New York, NY: Oxford University Press, 10.1093/MED/9780190497774.003.0001

[B68] Haxby J. V. Ungerleider L. G. Clark V. P. Schouten J. L. Hoffman E. A. Martin A. (1999). The effect of face inversion on activity in human neural systems for face and object peyrception. *Neuron* 22 189–199. 10.1016/S0896-6273(00)80690-X 10027301

[B69] Hayes D. J. Northoff G. (2012). Common brain activations for painful and non-painful aversive stimuli. *BMC Neurosci.* 13:60. 10.1186/1471-2202-13-60/FIGURES/3 PMC3464596 22676259

[B70] Hoffman E. A. Haxby J. V. (2000). Distinct representations of eye gaze and identity in the distributed human neural system for face perception. *Nat. Neurosci.* 3 80–84. 10.1038/71152 10607399

[B71] Hoshi H. Hirata Y. Fukasawa K. Kobayashi M. Shigihara Y. (2024). Oscillatory characteristics of resting-state magnetoencephalography reflect pathological and symptomatic conditions of cognitive impairment. *Front. Aging Neurosci.* 16:1273738. 10.3389/FNAGI.2024.1273738 38352236 PMC10861731

[B72] Hoshi H. Menninghaus W. (2018). The eye tracks the aesthetic appeal of sentences. *J. Vis.* 18 19–19. 10.1167/18.3.19 29677335

[B73] Iidaka T. (2014). Role of the fusiform gyrus and superior temporal sulcus in face perception and recognition: An empirical review. *Jpn. Psychol. Res.* 56 33–45. 10.1111/JPR.12018

[B74] Ishizu T. Zeki S. (2011). Toward a brain-based theory of beauty. *PLoS One* 6:e21852. 10.1371/JOURNAL.PONE.0021852 21755004 PMC3130765

[B75] Istók E. Brattico E. Jacobsen T. Krohn K. Müller M. Tervaniemi M. (2009). Aesthetic responses to music: A questionnaire study. *Music Sci.* 13 183–206. 10.1177/102986490901300201

[B76] Jacobsen T. Höfel L. (2003). Descriptive and evaluative judgment processes: Behavioral and electrophysiological indices of processing symmetry and aesthetics. *Cogn. Affect. Behav. Neurosci.* 3 289–299. 10.3758/CABN.3.4.289/METRICS 15040549

[B77] Jiang Y. Costello P. He S. (2007). Processing of invisible stimuli: Advantage of upright faces and recognizable words in overcoming interocular suppression. *Psychol. Sci.* 18 349–355. 10.1111/J.1467-9280.2007.01902.X 17470261

[B78] Jiang Y. He S. (2006). Cortical responses to invisible faces: Dissociating subsystems for facial-information processing. *Curr. Biol.* 16 2023–2029. 10.1016/J.CUB.2006.08.084 17055981

[B79] Kanai R. Carmel D. Bahrami B. Rees G. (2011). Structural and functional fractionation of right superior parietal cortex in bistable perception. *Curr. Biol.* 21 R106–R107. 10.1016/j.cub.2010.12.009 21300270 PMC3084447

[B80] Kanwisher N. McDermott J. Chun M. M. (1997). The fusiform face area: A module in human extrastriate cortex specialized for face perception. *J. Neurosci.* 17 4302–4311. 10.1523/JNEUROSCI.17-11-04302.1997 9151747 PMC6573547

[B81] Kawabata H. Zeki S. (2004). Neural correlates of beauty. *J. Neurophysiol.* 91 1699–1705. 10.1152/JN.00696.2003/ASSET/IMAGES/LARGE/Z9K0040437760007.JPEG 15010496

[B82] Kedia G. Mussweiler T. Mullins P. Linden D. E. J. (2014). The neural correlates of beauty comparison. *Soc. Cogn. Affect. Neurosci.* 9 681–688. 10.1093/SCAN/NST026 23508477 PMC4014099

[B83] Killgore W. D. S. Yurgelun-Todd D. A. (2004). Activation of the amygdala and anterior cingulate during nonconscious processing of sad versus happy faces. *Neuroimage* 21 1215–1223. 10.1016/J.NEUROIMAGE.2003.12.033 15050549

[B84] Kirk U. (2008). The neural basis of object-context relationships on aesthetic judgment. *PLoS One* 3:e3754. 10.1371/JOURNAL.PONE.0003754 19018279 PMC2582437

[B85] Knoop C. A. Wagner V. Jacobsen T. Menninghaus W. (2016). Mapping the aesthetic space of literature ‘from below.’. *Poetics* 56 35–49. 10.1016/J.POETIC.2016.02.001

[B86] Knyazev G. G. Slobodskoj-Plusnin J. Y. Bocharov A. V. (2009). Event-related delta and theta synchronization during explicit and implicit emotion processing. *Neuroscience* 164 1588–1600. 10.1016/J.NEUROSCIENCE.2009.09.057 19796666

[B87] Lang P. J. Bradley M. M. Cuthbert S. Greenwald M. Dhman A. Vaid D. (1997). International affective picture system (IAPS): Technical manual and affective ratings. *Univ. Florida Cent. Res. Psychophysiol. Gainesv.* 1:3.

[B88] Langlois J. H. Roggman L. A. Casey R. J. Ritter J. M. Rieser-Danner L. A. Jenkins V. Y. (1987). Infant preferences for attractive faces: Rudiments of a stereotype? *Dev. Psychol.* 23 363–369. 10.1037/0012-1649.23.3.363

[B89] LeDoux J. (1996). *The Emotional Brain: The Mysterious Underpinnings of Emotional Life.* New York, NY: Simon and Schuster.

[B90] Lin F. H. Witzel T. Ahlfors S. P. Stufflebeam S. M. Belliveau J. W. Hämäläinen M. S. (2006). Assessing and improving the spatial accuracy in MEG source localization by depth-weighted minimum-norm estimates. *Neuroimage* 31 160–171. 10.1016/J.NEUROIMAGE.2005.11.054 16520063

[B91] Little A. C. Jones B. C. Debruine L. M. (2011). Facial attractiveness: Evolutionary based research. *Philos. Trans. R. Soc. B Biol. Sci.* 366:1638. 10.1098/RSTB.2010.0404 21536551 PMC3130383

[B92] Logothetis N. K. Sheinberg D. L. (1996). Visual object recognition. *Annu. Rev. Neurosci.* 19 577–621. 10.1146/ANNUREV.NE.19.030196.003045 8833455

[B93] Machetanz K. Gallotti A. L. Leao Tatagiba M. T. Liebsch M. Trakolis L. Wang S. (2021). Time-frequency representation of motor evoked potentials in brain tumor patients. *Front. Neurol.* 11:633224. 10.3389/FNEUR.2020.633224/BIBTEX PMC7894199 33613426

[B94] Makeig S. Bell A. J. Jung T. P. Sejnowski T. J. (1996). ‘Independent component analysis of electroencephalographic data,’ in *Advances in Neural Information Processing Systems 8*, eds Touretzky D. Mozer M. Hasselmo M. (Cambridge, MA: MIT Press), 145–151.

[B95] Maris E. Oostenveld R. (2007). Nonparametric statistical testing of EEG- and MEG-data. *J. Neurosci. Methods* 164 177–190. 10.1016/j.jneumeth.2007.03.024 17517438

[B96] Martínez-Cagigal V. (2021). *Multiple Testing Toolbox.* Available online at: https://www.mathworks.com/matlabcentral/fileexchange/70604-multiple-testing-toolbox (accessed April 26, 2021).

[B97] Martín-Loeches M. Hernández-Tamames J. A. Martín A. Urrutia M. (2014). Beauty and ugliness in the bodies and faces of others: An fMRI study of person esthetic judgement. *Neuroscience* 277 486–497. 10.1016/J.NEUROSCIENCE.2014.07.040 25086316

[B98] Mcbrearty S. Brooks A. S. (2000). The revolution that wasn’t: A new interpretation of the origin of modern human behavior. *J. Hum. Evol.* 39 453–563. 10.1006/JHEV.2000.0435 11102266

[B99] Montoya R. M. Horton R. S. Vevea J. L. Citkowicz M. Lauber E. A. (2017). A re-examination of the mere exposure effect: The influence of repeated exposure on recognition, familiarity, and liking. *Psychol. Bull.* 143 459–498. 10.1037/BUL0000085 28263645

[B100] Mudrik L. Deouell L. Y. (2022). Neuroscientific evidence for processing without awareness. *Annu. Rev. Neurosci.* 45 403–423. 10.1146/ANNUREV-NEURO-110920-033151 35803585

[B101] Muñoz F. Martín-Loeches M. (2015). Electrophysiological brain dynamics during the esthetic judgment of human bodies and faces. *Brain Res.* 1594 154–164. 10.1016/J.BRAINRES.2014.10.061 25451119

[B102] Nakamura K. Kawashima R. Nagumo S. Ito K. Sugiura M. Kato T. (1998). Neuroanatomical correlates of the assessment of facial attractiveness. *Neuroreport* 9 753–757. 10.1097/00001756-199803090-00035 9559951

[B103] Natu V. O’Toole A. J. (2011). The neural processing of familiar and unfamiliar faces: A review and synopsis. *Br. J. Psychol.* 102 726–747. 10.1111/J.2044-8295.2011.02053.X 21988381

[B104] Oostenveld R. Fries P. Maris E. Schoffelen J.-M. (2011). FieldTrip: Open source software for advanced analysis of MEG, EEG, and invasive electrophysiological data. *Comput. Intell. Neurosci.* 2011:156869. 10.1155/2011/156869 21253357 PMC3021840

[B105] Parkkonen L. Andersson J. Hämäläinen M. Hari R. (2008). Early visual brain areas reflect the percept of an ambiguous scene. *Proc. Natl. Acad. Sci. U. S. A.* 105 20500–20504. 10.1073/PNAS.0810966105 19074267 PMC2602606

[B106] Pesciarelli F. Leo I. Serafini L. (2021). Electrophysiological correlates of unconscious processes of race. *Sci. Rep.* 11 1–12. 10.1038/s41598-021-91133-2 34079021 PMC8172900

[B107] Pessoa L. Adolphs R. (2010). Emotion processing and the amygdala: From a ‘low road’ to ‘many roads’ of evaluating biological significance. *Nat. Rev. Neurosci.* 11 773–782. 10.1038/nrn2920 20959860 PMC3025529

[B108] Piai V. Roelofs A. Van Der Meij R. (2012). Event-related potentials and oscillatory brain responses associated with semantic and Stroop-like interference effects in overt naming. *Brain Res.* 1450 87–101. 10.1016/J.BRAINRES.2012.02.050 22424790

[B109] Pinker S. (1997). *How the Mind Works.* New York, NY: W.W. Norton & Company.

[B110] Pinker S. Fodor J. (2005). So how does the mind work? *Mind Lang.* 20 1–24. 10.1111/J.0268-1064.2005.00274.X

[B111] Pitcher D. Dilks D. D. Saxe R. R. Triantafyllou C. Kanwisher N. (2011). Differential selectivity for dynamic versus static information in face-selective cortical regions. *Neuroimage* 56 2356–2363. 10.1016/J.NEUROIMAGE.2011.03.067 21473921

[B112] Pitcher D. Japee S. Rauth L. Ungerleider L. G. (2017). The superior temporal sulcus is causally connected to the Amygdala: A combined TBS-fMRI Study. *J. Neurosci.* 37 1156–1161. 10.1523/JNEUROSCI.0114-16.2016 28011742 PMC5296794

[B113] Popov T. Oostenveld R. Schoffelen J. M. (2018). FieldTrip made easy: An analysis protocol for group analysis of the auditory steady state brain response in time, frequency, and space. *Front. Neurosci.* 12:402658. 10.3389/FNINS.2018.00711/BIBTEX PMC6189392 30356712

[B114] Rajimehr R. Young J. C. Tootell R. B. H. (2009). An anterior temporal face patch in human cortex, predicted by macaque maps. *Proc. Natl. Acad. Sci. U. S. A.* 106 1995–2000. 10.1073/PNAS.0807304106/SUPPL_FILE/0807304106SI.PDF 19179278 PMC2632713

[B115] Reber R. Schwarz N. Winkielman P. (2004). Processing fluency and aesthetic pleasure: is beauty in the perceiver’s processing experience? *Personal. Soc. Psychol. Rev.* 8 364–382. 10.1207/S15327957PSPR0804_3 15582859

[B116] Reber R. Winkielman P. Schwarz N. (1998). Effects of perceptual fluency on affective judgments. *Psychol. Sci.* 9 45–48. 10.1111/1467-9280.00008

[B117] Rhodes G. (2005). The evolutionary psychology of facial beauty. *Annu. Rev. Psychol.* 57 199–226. 10.1146/ANNUREV.PSYCH.57.102904.190208 16318594

[B118] Rothenberg D. (2011). *Survival of the Beautiful: Art, Science, and Evolution.* London: Bloomsbury Publishing.

[B119] Rusch H. Voland E. (2013). Evolutionary aesthetics: An introduction to key concepts and current issues. *Aisthesis* 6 113–133. 10.13128/AISTHESIS-13773

[B120] Sarasso P. Ronga I. Kobau P. Bosso T. Artusio I. Ricci R. (2020). Beauty in mind: Aesthetic appreciation correlates with perceptual facilitation and attentional amplification. *Neuropsychologia* 136:107282. 10.1016/J.NEUROPSYCHOLOGIA.2019.107282 31770549

[B121] Schubring D. Schupp H. T. (2021). Emotion and brain oscillations: High arousal is associated with decreases in alpha- and lower beta-band power. *Cereb. Cortex* 31 1597–1608. 10.1093/CERCOR/BHAA312 33136146

[B122] Sekihara K. Kawabata Y. Ushio S. Sumiya S. Kawabata S. Adachi Y. (2016). Dual signal subspace projection (DSSP): A novel algorithm for removing large interference in biomagnetic measurements. *J. Neural Eng.* 13:036007. 10.1088/1741-2560/13/3/036007 27064933 PMC6287966

[B123] Sergerie K. Chochol C. Armony J. L. (2008). The role of the amygdala in emotional processing: A quantitative meta-analysis of functional neuroimaging studies. *Neurosci. Biobehav. Rev.* 32 811–830. 10.1016/J.NEUBIOREV.2007.12.002 18316124

[B124] Singer T. Critchley H. D. Preuschoff K. (2009). A common role of insula in feelings, empathy and uncertainty. *Trends Cogn. Sci.* 13 334–340. 10.1016/j.tics.2009.05.001 19643659

[B125] Somerville L. H. Kim H. Johnstone T. Alexander A. L. Whalen P. J. (2004). Human amygdala responses during presentation of happy and neutral faces: Correlations with state anxiety. *Biol. Psychiatry* 55 897–903. 10.1016/J.BIOPSYCH.2004.01.007 15110733

[B126] Starr G. G. (2023). Aesthetic experience models human learning. *Front. Hum. Neurosci.* 17:1146083. 10.3389/FNHUM.2023.1146083/BIBTEX PMC10185790 37200953

[B127] Stegemöller E. L. Ferguson T. D. Zaman A. Hibbing P. Izbicki P. Krigolson O. E. (2021). Finger tapping to different styles of music and changes in cortical oscillations. *Brain Behav.* 11:e2324. 10.1002/BRB3.2324 34423594 PMC8442589

[B128] Stein T. Hebart M. N. Sterzer P. (2011). Breaking continuous flash suppression: A new measure of unconscious processing during interocular suppression? *Front. Hum. Neurosci.* 5:13980. 10.3389/FNHUM.2011.00167/BIBTEX PMC3243089 22194718

[B129] Stein T. Sterzer P. Peelen M. V. (2012). Privileged detection of conspecifics: Evidence from inversion effects during continuous flash suppression. *Cognition* 125 64–79. 10.1016/J.COGNITION.2012.06.005 22776239

[B130] Stelter M. Schweinberger S. R. (2023). Understanding the mechanisms underlying the other-‘race’ effect: An attempt at integrating different perspectives. *Br. J. Psychol.* 114 1–9. 10.1111/BJOP.12615 36583346

[B131] Tadel F. Baillet S. Mosher J. C. Pantazis D. Leahy R. M. (2011). Brainstorm: A user-friendly application for MEG/EEG analysis. *Comput. Intell. Neurosci.* 2011 1–13. 10.1155/2011/879716 21584256 PMC3090754

[B132] Tamietto M. De Gelder B. (2010). Neural bases of the non-conscious perception of emotional signals. *Nat. Rev. Neurosci.* 11 697–709. 10.1038/nrn2889 20811475

[B133] Thornhill R. Gangestad S. W. Thornhill R. Gangestad S. W. Thornhill R. Gangestad S. W. (1999). Facial attractiveness. *Trends Cogn. Sci.* 3 452–460. 10.1016/S1364-6613(99)01403-5 10562724

[B134] Tinbergen N. (1963). On aims and methods of Ethology. *Z. Tierpsychol.* 20 410–433. 10.1111/J.1439-0310.1963.TB01161.X

[B135] Tong F. (2003). Primary visual cortex and visual awareness. *Nat. Rev. Neurosci.* 4 219–229. 10.1038/nrn1055 12612634

[B136] Tsao D. Y. Schweers N. Moeller S. Freiwald W. A. (2008). Patches of face-selective cortex in the macaque frontal lobe. *Nat. Neurosci.* 11 877–879. 10.1038/nn.2158 18622399 PMC8123225

[B137] Tsuchiya N. Koch C. (2004). Continuous flash suppression. *J. Vis.* 4:61. 10.1167/4.8.61 17132078

[B138] Tsuchiya N. Koch C. (2005). Continuous flash suppression reduces negative afterimages. *Nat. Neurosci.* 8 1096–1101. 10.1038/nn1500 15995700

[B139] Tsukiura T. Cabeza R. (2011). Shared brain activity for aesthetic and moral judgments: implications for the Beauty-is-Good stereotype. *Soc. Cogn. Affect. Neurosci.* 6 138–148. 10.1093/SCAN/NSQ025 20231177 PMC3023089

[B140] Uddin L. Q. Nomi J. S. Hébert-Seropian B. Ghaziri J. Boucher O. (2017). Structure and function of the human insula. *J. Clin. Neurophysiol.* 34:300. 10.1097/WNP.0000000000000377 28644199 PMC6032992

[B141] Van De Cruys S. Frascaroli J. Friston K. (2024). Order and change in art: Towards an active inference account of aesthetic experience. *Philos. Trans. R. Soc. B.* 379:20220411. 10.1098/RSTB.2022.0411 38104600 PMC10725768

[B142] van der Wel P. van Steenbergen H. (2018). Pupil dilation as an index of effort in cognitive control tasks: A review. *Psychon. Bull. Rev.* 25 2005–2015. 10.3758/S13423-018-1432-Y/METRICS 29435963 PMC6267528

[B143] van Gaal S. Naccache L. Meuwese J. D. I. van Loon A. M. Leighton A. H. Cohen L. (2014). Can the meaning of multiple words be integrated unconsciously? *Philos. Trans. R. Soc. B Biol. Sci.* 369:20130212. 10.1098/RSTB.2013.0212 24639583 PMC3965166

[B144] Vartanian O. Goel V. (2004). Neuroanatomical correlates of aesthetic preference for paintings. *Neuroreport* 15 893–897. 10.1097/00001756-200404090-00032 15073538

[B145] Vessel E. A. Maurer N. Denker A. H. Starr G. G. (2018). Stronger shared taste for natural aesthetic domains than for artifacts of human culture. *Cognition* 179 121–131. 10.1016/J.COGNITION.2018.06.009 29936343

[B146] Vessel E. A. Stahl J. Maurer N. Denker A. Starr G. G. (2014). Personalized visual aesthetics. *Hum. Vis. Electron. Imaging XIX* 25:9014. 10.1117/12.2043126

[B147] Vialatte F. B. Maurice M. Dauwels J. Cichocki A. (2010). Steady-state visually evoked potentials: Focus on essential paradigms and future perspectives. *Prog. Neurobiol.* 90 418–438. 10.1016/J.PNEUROBIO.2009.11.005 19963032

[B148] Voland E. Grammer K. (2003). *Evolutionary Aesthetics.* Berlin: Springer-Verlag.

[B149] Wassiliwizky E. Menninghaus W. (2021). Why and how should cognitive science care about aesthetics? *Trends Cogn. Sci.* 25 437–449. 10.1016/J.TICS.2021.03.008 33810983

[B150] Wendt J. Lotze M. Weike A. I. Hosten N. Hamm A. O. (2008). Brain activation and defensive response mobilization during sustained exposure to phobia-related and other affective pictures in spider phobia. *Psychophysiology* 45 205–215. 10.1111/J.1469-8986.2007.00620.X 17995911

[B151] Willenbockel V. Sadr J. Fiset D. Horne G. O. Gosselin F. Tanaka J. W. (2010). Controlling low-level image properties: The SHINE toolbox. *Behav. Res. Methods* 42 671–684. 10.3758/BRM.42.3.671/METRICS 20805589

[B152] Winston J. S. O’Doherty J. Kilner J. M. Perrett D. I. Dolan R. J. (2007). Brain systems for assessing facial attractiveness. *Neuropsychologia* 45 195–206. 10.1016/J.NEUROPSYCHOLOGIA.2006.05.009 16828125

[B153] Yoo J. Jasko K. Winkielman P. (2024). Fluency, prediction and motivation: How processing dynamics, expectations and epistemic goals shape aesthetic judgements. *Philos. Trans. R. Soc. B.* 379:20230326. 10.1098/RSTB.2023.0326 38104614 PMC10725759

[B154] Zeki S. (2004). The neurology of ambiguity. *Conscious. Cogn.* 13 173–196. 10.1016/J.CONCOG.2003.10.003 14990252

[B155] Zhu W. Drewes J. Melcher D. (2016). Time for awareness: The influence of temporal properties of the mask on continuous flash suppression effectiveness. *PLoS One* 11:e0159206. 10.1371/JOURNAL.PONE.0159206 27416317 PMC4945020

