# Supplementary material for: Binocularly suppressed stimuli induce brain activities related to aesthetic emotions
Source: Front Neurosci. 2024 May 24;18:1339479. doi: 10.3389/fnins.2024.1339479 (PMC11159128; doi:10.3389/fnins.2024.1339479)
Supplement: Supplementary file 1 [file Data_Sheet_1.docx]

Supplementary Material

Binocularly suppressed stimuli induce brain activities related to aesthetic emotions

Hideyuki Hoshi^*^, Akira Ishii, Yoshihito Shigihara, and Takahiro Yoshikawa

*** Correspondence:**

Hideyuki Hoshi
heurekaesthem.avir@gmail.com

# Stimulus information

**Table S1. List of paintings used for facial stimuli.**

| Painter | Style /  Art movement | ID | Title (year) - English translation | Sex |
| --- | --- | --- | --- | --- |
| Hans Holbein the Younger | Northern Renaissance | 01 | Christina of Denmark (1538) | Woman |
|  |  | 02 | Lais Corinthiaca (1526) | Woman |
|  |  | 03 | Venus and Amor (1524-1525) | Woman |
|  |  | 04 | Portrait of a Member of the Wedigh Family (1532) | Man |
|  |  | 05 | Portrait of Sir Thomas More (1527) | Man |
| Raphael | High Renaissance | 06 | The Portrait of a Young Woman (La Fornarina) (1518-1520) | Woman |
|  |  | 07 | The Veiled Woman, or La Donna Velata (1516) | Woman |
|  |  | 08 | Portrait of a Young Man (1515) | Man |
|  |  | 09 | Portrait of Bindo Altoviti (1512-1515) | Man |
|  |  | 10 | Self Portrait (1506) | Man |
| Frans Hals | Baroque, Dutch Golden Age | 11 | Portrait of a Woman (1638) | Woman |
|  |  | 12 | Portrait of a woman, possibly Sara Wolphaerts van Diemen (1635) | Woman |
|  |  | 13 | Portrait of a woman (1644) | Woman |
|  |  | 14 | Portrait of Jean de la Chambre at the age of 33 (1638) | Man |
|  |  | 15 | Vincent Laurensz. van der Vinne. (1655-1660) | Man |
| Thomas Lawrence | Rococo, Romanticism | 16 | Head of a Girl (?) | Woman |
|  |  | 17 | Lady Caroline Lamb (1827) | Woman |
|  |  | 18 | Princess Lieven (?) | Woman |
|  |  | 19 | Sir William Grant (1802) | Man |
|  |  | 20 | George Granville Leveson-Gower (1800) | Man |
| Pierre-Auguste Renoir | Impressionism | 21 | Romaine Lascaux (1864) | Woman |
|  |  | 22 | Woman with a rose (1875-1876) | Woman |
|  |  | 23 | Eugene Murer (1877) | Man |
|  |  | 24 | Portrait of Victor Chocquet (1875) | Man |
|  |  | 25 | Stephane Mallarme (1892) | Man |
| Camille Pissarro | Impressionism, Neo-Impressionism | 26 | Portrait of Georges (1880) | Woman |
|  |  | 27 | Paul Emile Pissarro (1890) | Woman |
|  |  | 28 | Portrait of Eugene Murer (1878) | Man |
|  |  | 29 | Portrait of Monsieur Louis Estruc (1874) | Man |
|  |  | 30 | Portrait of the Artist's Son, Ludovic Rudolphe (1888) | Man |
| Amedeo Modigliani | Expressionism | 31 | Head of a Young Girl (1916) | Woman |
|  |  | 32 | Paulette Jourdain (1919) | Woman |
|  |  | 33 | Leopold Zborowski (1918) | Man |
|  |  | 34 | The Boy (1918) | Man |
|  |  | 35 | Young Man (Student) (1919) | Man |
| Juan Gris | Cubism | 36 | Seated Woman (1917) | Woman |
|  |  | 37 | Portrait of Madame Josette Gris (1916) | Woman |
|  |  | 38 | Portrait of the Artist’s Mother (1912) | Woman |
|  |  | 39 | Portrait of Pablo Picasso (1912) | Man |
|  |  | 40 | Man from Touraine (1918) | Man |
| Paul Klee | Expressionism, Abstract Art, Surrealism | 41 | Woman in Peasant Dress (1940) | Woman |
|  |  | 42 | Evening shows (1935) | Unknown |
|  |  | 43 | Gauze (1940) | Unknown |
|  |  | 44 | Pierrot Lunaire (1924) | Unknown |
|  |  | 45 | Senecio (1922) | Unknown |
| Josef Capek | Cubism | 46 | Hlava divky (Slepa, Hlava zeny) (1916) - Girl's Head (Blind, Woman's Head) | Woman |
|  |  | 47 | Muz s knirkem (1915) - Man with a mustache | Man |
|  |  | 48 | Sportovec s cervenou hvezdou (1915) - Athlete with a red star | Man |
|  |  | 49 | Hlava (1915) - Head | Unknown |
|  |  | 50 | Detektiv (1915-1916) - Detective | Unknown |

**Table S2. Results of replication test of the pre-rating scores.**

| ID | Interval  (days) | BIOL | | | OBJ | | | FACE | | | LIKE | | | BEAU | | |
| --- | --- | --- | --- | --- | --- | --- | --- | --- | --- | --- | --- | --- | --- | --- | --- | --- |
|  |  | *rho* | *CI*  (LL) | *CI*  (UL) | *rho* | *CI*  (LL) | *CI*  (UL) | *rho* | *CI*  (LL) | *CI*  (UL) | *rho* | *CI*  (LL) | *CI*  (UL) | *rho* | *CI*  (LL) | *CI*  (UL) |
| X0001 | 409 | 0.762 | 0.559 | 0.874 | 0.794 | 0.601 | 0.898 | 0.786 | 0.596 | 0.881 | 0.616 | 0.359 | 0.787 | 0.703 | 0.492 | 0.826 |
| X0002 | 598 | 0.849 | 0.578 | 0.945 | 0.899 | 0.785 | 0.955 | 0.921 | 0.825 | 0.969 | 0.509 | 0.242 | 0.711 | 0.716 | 0.517 | 0.852 |
| X0003 | 475 | 0.639 | 0.386 | 0.811 | 0.518 | 0.269 | 0.691 | 0.712 | 0.481 | 0.847 | 0.426 | 0.114 | 0.664 | 0.691 | 0.528 | 0.805 |

BIOL, *biologi-ness* score; OBJ, *object saliency* score; FACE, *facial saliency* score; LIKE; *liking* score; BEAU, *beauty* score; *rho*, Spearman’s coefficient (test-retest correlation); *CI* (LL), 95% bootstrap confidence interval (lower limit) of *rho*; *CI* (UL), 95% bootstrap confidence interval (upper limit) of *rho*.

**
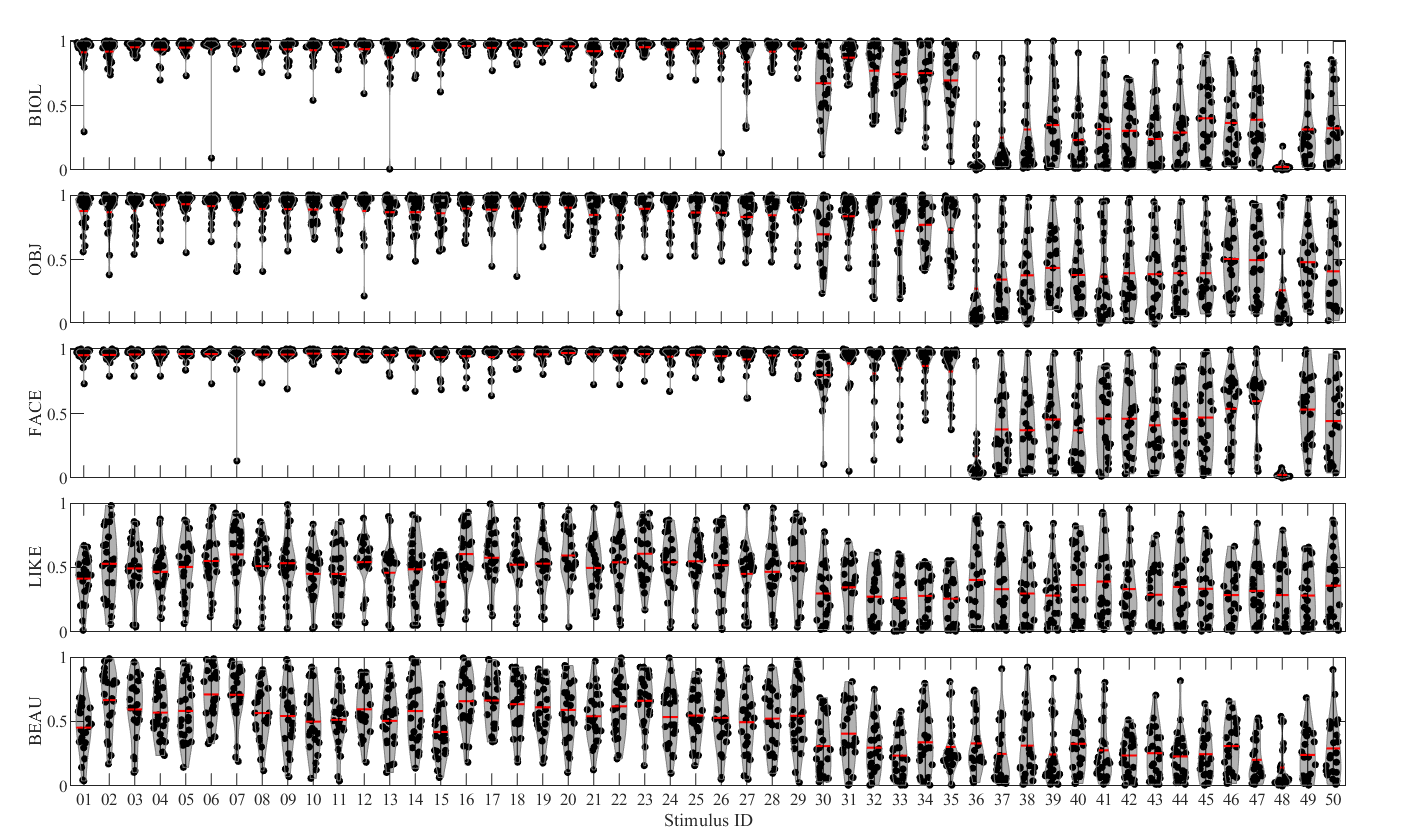
**

**Figure S1. Summary of the pre-rating scores.** Violin plot was drawn for each score (row) and for each stimulus (column) using data from all participants (N = 23). A red line indicates the mean of the plot. BIOL, *biologi-ness* score; OBJ, *object saliency* score; FACE, *facial saliency* score; LIKE; *liking* score; BEAU, *beauty* score.

**
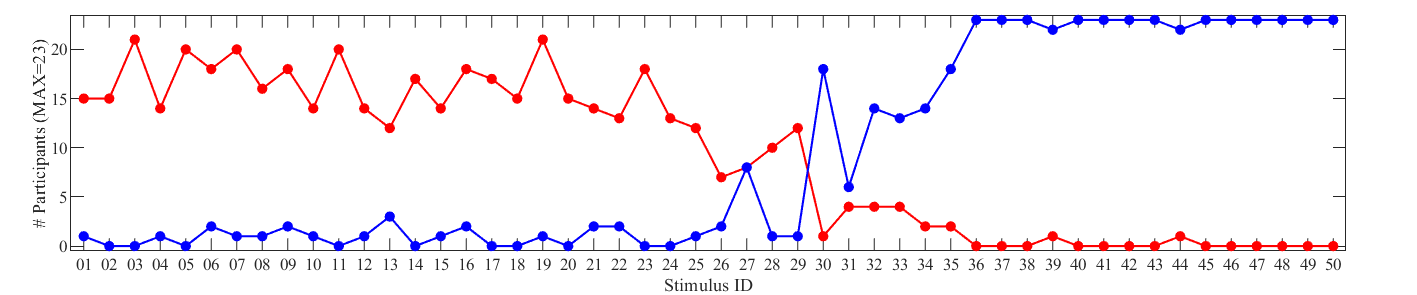
**

**Figure S2. Summary of the stimulus selection.** Red line represents the number of participants for whom the stimulus was selected as the biological stimuli (with the highest *biologi-ness* rating given by each participant). Blue line represents the number of participants for whom the stimulus was selected as the non-biological stimuli (with the lowest *biologi-ness* rating given by each participant).

# Results of behavioural data analysis

**
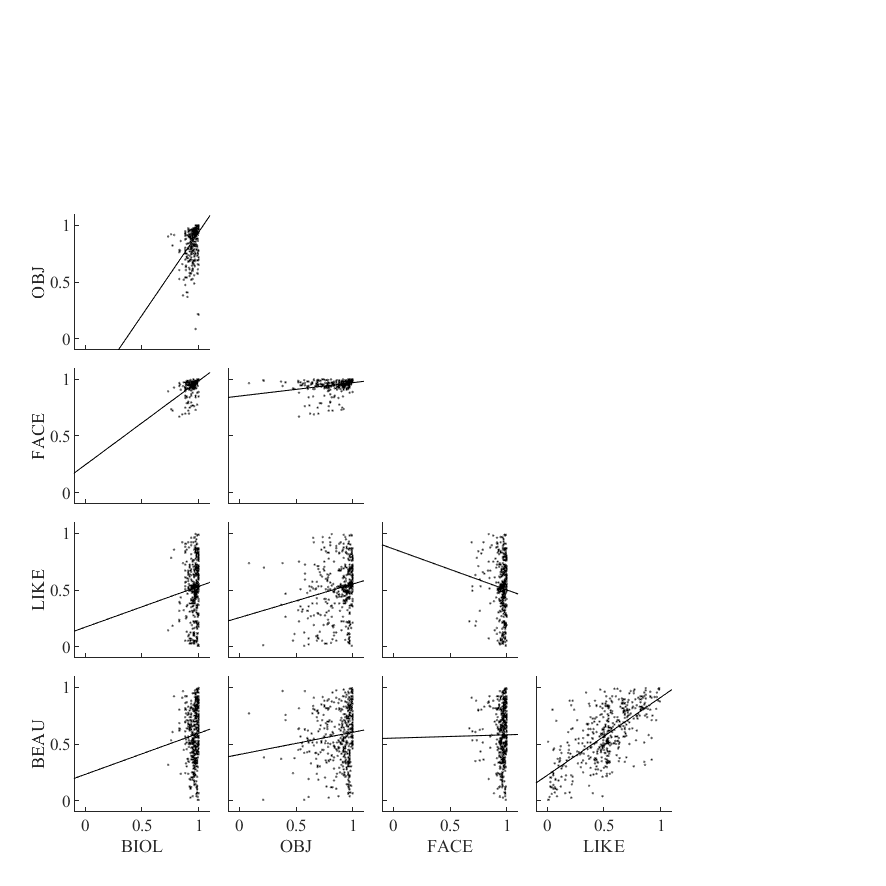
**

**Figure S3. Correlations between pre-rating scores in the biological category.** A least square line was superimposed on each scatterplot. BIOL, *biologi-ness* score; OBJ, *object saliency* score; FACE, *facial saliency* score; LIKE; *liking* score; BEAU, *beauty* score.

**Table S3. Correlations between pre-rating scores in the biological category.**

|  | BIOL | | OBJ | | FACE | | LIKE | |
| --- | --- | --- | --- | --- | --- | --- | --- | --- |
|  | *rho* | *P* (FDR) | *rho* | *P* (FDR) | *rho* | *P* (FDR) | *rho* | *P* (FDR) |
| OBJ | 0.171 | 0.001* |  |  |  |  |  |  |
| FACE | 0.144 | 0.008* | 0.145 | 0.009* |  |  |  |  |
| LIKE | 0.154 | 0.008* | 0.177 | <0.001* | 0.149 | 0.041* |  |  |
| BEAU | 0.130 | 0.019* | 0.245 | <0.001* | 0.103 | 0.084 | 0.456 | <0.001* |

An asterisk (*) indicates a significant correlation. *rho*, Spearman’s coefficient, averaged across bootstrap iterations; *P* (FDR), *P*-values controlled for the false discovery rate; BIOL, *biologi-ness* score; OBJ, *object saliency* score; FACE, *facial saliency* score; LIKE; *liking* score; BEAU, *beauty* score.

**
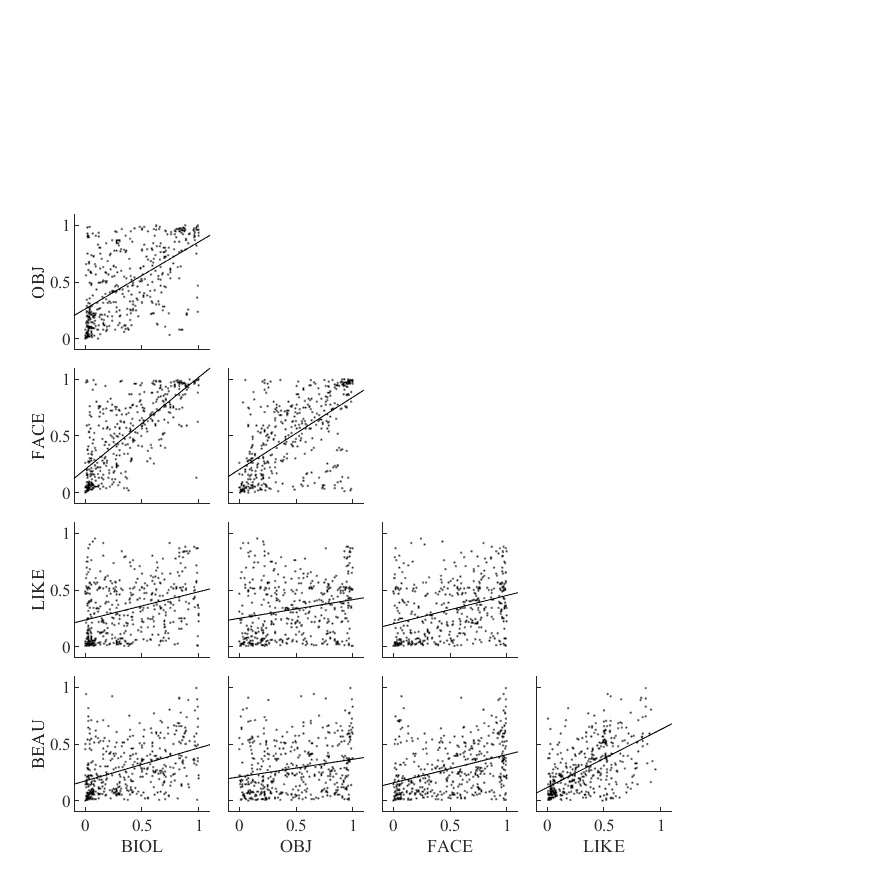
**

**Figure S4. Correlations between pre-rating scores in the non-biological category.** A least square line was superimposed on each scatterplot. BIOL, *biologi-ness* score; OBJ, *object saliency* score; FACE, *facial saliency* score; LIKE; *liking* score; BEAU, *beauty* score.

**Table S4. Correlations between pre-rating scores in the non-biological category.**

|  | BIOL | | OBJ | | FACE | | LIKE | |
| --- | --- | --- | --- | --- | --- | --- | --- | --- |
|  | *rho* | *P* (FDR) | *rho* | *P* (FDR) | *rho* | *P* (FDR) | *rho* | *P* (FDR) |
| OBJ | 0.626 | <0.001* |  |  |  |  |  |  |
| FACE | 0.691 | <0.001* | 0.652 | <0.001* |  |  |  |  |
| LIKE | 0.122 | 0.090 | 0.121 | 0.085 | 0.115 | 0.090 |  |  |
| BEAU | 0.280 | <0.001* | 0.239 | <0.001* | 0.261 | <0.001* | 0.371 | <0.001* |

An asterisk (*) indicates a significant correlation. *rho*, Spearman’s coefficient, averaged across bootstrap iterations; *P* (FDR), *P*-values controlled for the false discovery rate; BIOL, *biologi-ness* score; OBJ, *object saliency* score; FACE, *facial saliency* score; LIKE; *liking* score; BEAU, *beauty* score.

# Results of all data analysis (without categorisation)

**Table S5. (All data) Summary of the pre-rating data.**

|  |  | *M* | *SE* | *CI* (LL) | *CI* (UL) |
| --- | --- | --- | --- | --- | --- |
| BIOL |  | 0.716 | 0.019 | 0.683 | 0.757 |
| OBJ |  | 0.718 | 0.028 | 0.662 | 0.770 |
| FACE |  | 0.775 | 0.020 | 0.735 | 0.811 |
| LIKE |  | 0.431 | 0.031 | 0.362 | 0.483 |
| BEAU |  | 0.448 | 0.031 | 0.383 | 0.503 |

BIOL, *biologi-ness* score; OBJ, *object saliency* score; FACE, *facial saliency* score; LIKE, *liking* score; BEAU, *beauty* score; *M*, mean; *SE*, standard error; *CI* (LL), 95% bootstrap confidence interval (lower limit); *CI* (UL), 95% bootstrap confidence interval (upper limit)

**
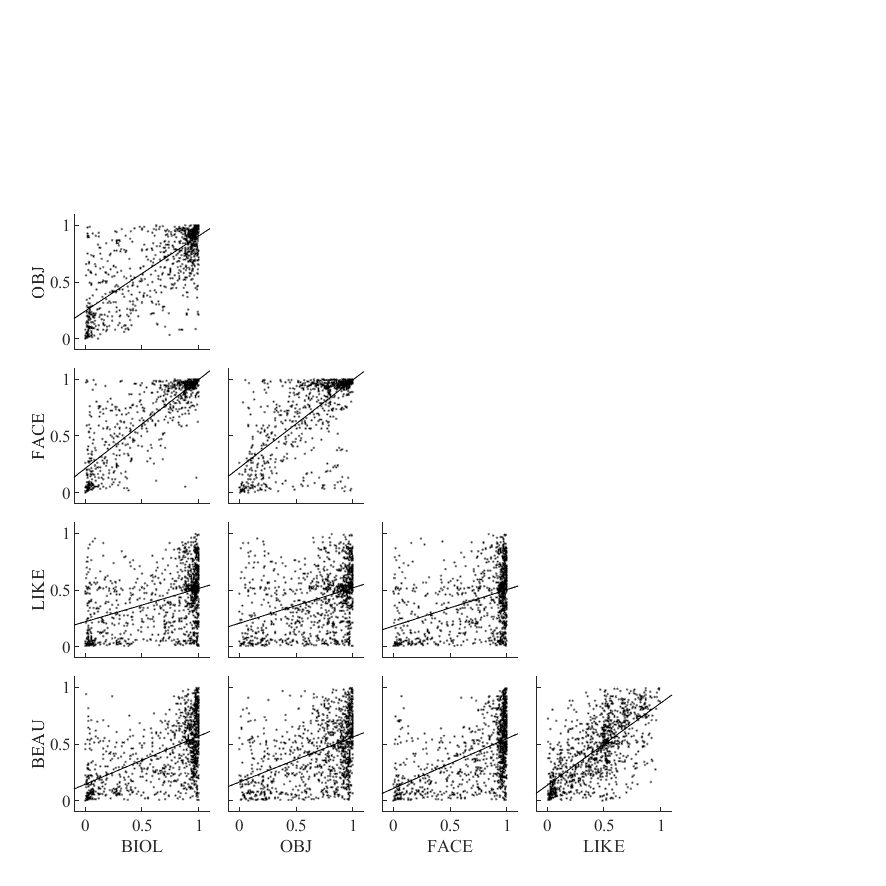
**

**Figure S5. (All data) Correlations between pre-rating scores.** A least square line was superimposed on each scatterplot. BIOL, *biologi-ness* score; OBJ, *object saliency* score; FACE, *facial saliency* score; LIKE; *liking* score; BEAU, *beauty* score.

**Table S6. (All data) Correlations between pre-rating scores.**

|  | BIOL | | OBJ | | FACE | | LIKE | |
| --- | --- | --- | --- | --- | --- | --- | --- | --- |
|  | *rho* | *P* (FDR) | *rho* | *P* (FDR) | *rho* | *P* (FDR) | *rho* | *P* (FDR) |
| OBJ | 0.695 | <0.001* |  |  |  |  |  |  |
| FACE | 0.737 | <0.001* | 0.684 | <0.001* |  |  |  |  |
| LIKE | 0.407 | <0.001* | 0.409 | <0.001* | 0.379 | <0.001* |  |  |
| BEAU | 0.587 | <0.001* | 0.576 | <0.001* | 0.553 | <0.001* | 0.561 | <0.001* |

An asterisk (*) indicates a significant correlation. *rho*, Spearman’s coefficient, averaged across bootstrap iterations; *P* (FDR), *P*-values controlled for the false discovery rate; BIOL, *biologi-ness* score; OBJ, *object saliency* score; FACE, *facial saliency* score; LIKE; *liking* score; BEAU, *beauty* score.

**
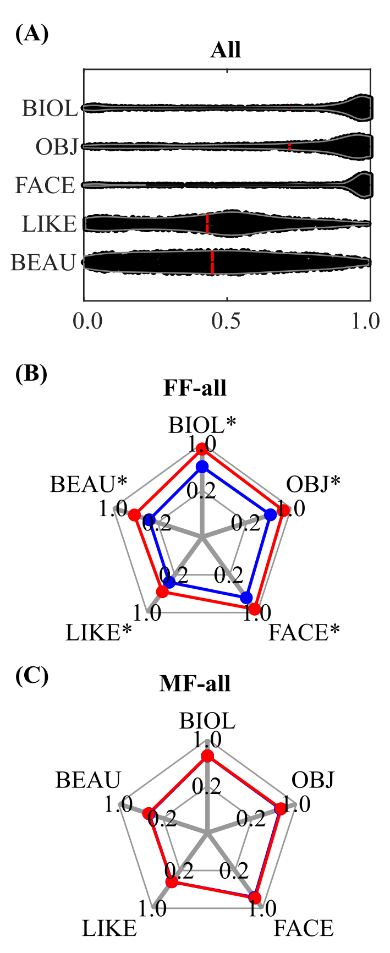
**

**Figure S6. (All data)** Results of behavioural data analysis for all data. **(A)** A violin plot visualising the distribution of each pre-rating score, with the mean value represented by a red line. **(B)** A spider plot contrasting the mean of each pre-rating score in the FF condition, in which participants responded as ‘yes’ (red: ‘beauty’) and ‘no’ (blue: ‘non-beauty’) to the Q2 (‘beauty?’ question), respectively. **(C)** A spider plot contrasting the mean of each pre-rating score in the MF condition, in which participants responded as ‘yes’ (red: ‘beauty’) and ‘no’ (blue: ‘non-beauty’) to the Q2 (‘beauty?’ question), respectively. An asterisk (*) indicates a significant difference in pre-ratings scores between ‘yes’ (‘beauty’ response) and ‘no’ (‘non-beauty’ response) trials. FF, face-face condition; MF, Mondrian-face condition; BIOL, *biologi-ness* rating; OBJ, *object saliency* rating; FACE, *facial saliency* rating; LIKE; *liking* rating; BEAU, *beauty* rating.

**Table S7. (All data) Summary of the relationships between pre-rating scores and response data.**

|  |  |  | Beauty trials | | | |  | Non-beauty trials | | | |  |  |
| --- | --- | --- | --- | --- | --- | --- | --- | --- | --- | --- | --- | --- | --- |
|  |  |  | *M* | *SE* | *CI* (LL) | *CI* (UL) |  | *M* | *SE* | *CI* (LL) | *CI* (UL) |  | *P* (FDR) |
| (A) FF | | | | | | | | | | | | | |
|  | All data | | | | | | | | | | | | |
|  |  | %Trials | 0.346 | 0.035 |  |  |  | 0.654 | 0.035 |  |  |  |  |
|  |  | BIOL | 0.906 | 0.017 | 0.865 | 0.932 |  | 0.604 | 0.033 | 0.541 | 0.666 |  | <0.001* |
|  |  | OBJ | 0.862 | 0.022 | 0.814 | 0.900 |  | 0.628 | 0.037 | 0.556 | 0.700 |  | <0.001* |
|  |  | FACE | 0.928 | 0.014 | 0.893 | 0.948 |  | 0.686 | 0.031 | 0.615 | 0.737 |  | <0.001* |
|  |  | LIKE | 0.559 | 0.031 | 0.494 | 0.612 |  | 0.358 | 0.031 | 0.296 | 0.414 |  | <0.001* |
|  |  | BEAU | 0.619 | 0.031 | 0.552 | 0.671 |  | 0.353 | 0.028 | 0.298 | 0.406 |  | <0.001* |
| (B) MF | | | | | | | | | | | | | |
|  | All data | | | | | | | | | | | | |
|  |  | %Trials | 0.324 | 0.049 |  |  |  | 0.676 | 0.049 |  |  |  |  |
|  |  | BIOL | 0.716 | 0.028 | 0.662 | 0.770 |  | 0.706 | 0.025 | 0.661 | 0.757 |  | 0.782 |
|  |  | OBJ | 0.724 | 0.033 | 0.661 | 0.788 |  | 0.699 | 0.035 | 0.628 | 0.765 |  | 0.389 |
|  |  | FACE | 0.784 | 0.026 | 0.730 | 0.829 |  | 0.757 | 0.025 | 0.708 | 0.803 |  | 0.389 |
|  |  | LIKE | 0.437 | 0.029 | 0.377 | 0.487 |  | 0.448 | 0.037 | 0.365 | 0.511 |  | 0.678 |
|  |  | BEAU | 0.460 | 0.029 | 0.405 | 0.517 |  | 0.454 | 0.038 | 0.377 | 0.524 |  | 0.782 |

An asterisk (*) indicates a significant difference between the Beauty vs. Non-beauty trials. FF, face-face condition; MF, Mondrian-face condition; MB, Mondrian-background condition; BIOL, *biologi-ness* score; OBJ, *object saliency* score; FACE, *facial saliency* score; LIKE, *liking* score; BEAU, *beauty* score; *M*, mean; *SE*, standard error; *CI* (LL), 95% bootstrap confidence interval (lower limit); *CI* (UL), 95% bootstrap confidence interval (upper limit); *P* (FDR), *P*-values controlled for the false discovery rate.

**
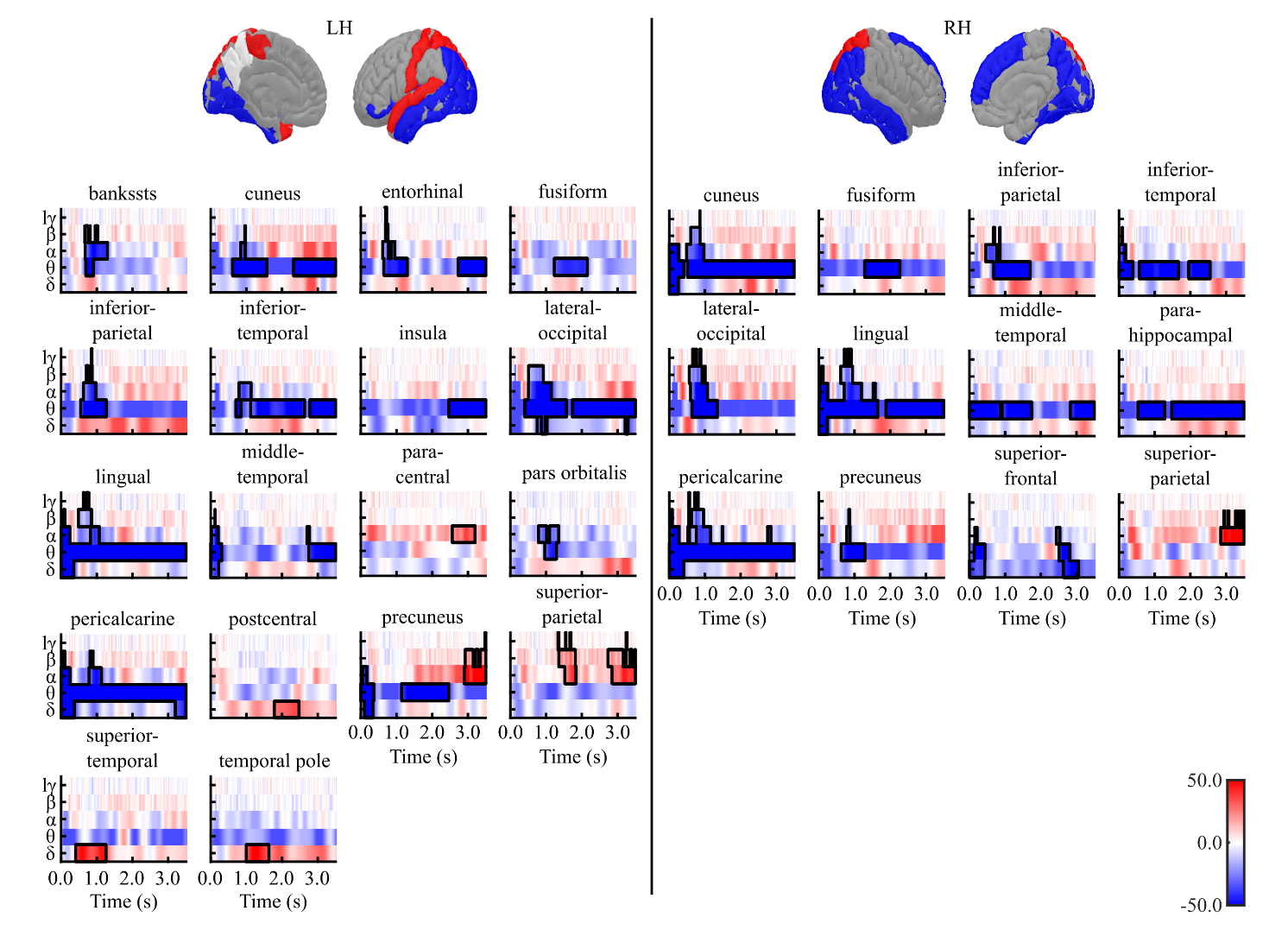
**

**Figure S7. (All data)** Results of the cluster-based permutation tests between the FF-all vs. MF-all conditions. All TF images are scaled equally, with significant clusters emphasised using black bold lines. The regions (ROIs) in the template brain images were coloured red when any positive clusters were found in the ROI, blue when any negative clusters were found in the ROI, or white when both positive and negative clusters were found in the ROI. The results for hγ band are not displayed, because no significant clusters were found in the band. FF, face-face condition; MF, Mondrian-face condition; LH, left hemisphere; RH, right hemisphere; lγ, low-gamma; hγ, high-gamma.

**Table S8. (All data) Results of the cluster-based permutation tests for TF data between the FF-all and MF-all conditions.**

|  | Cluster | | | Peak | | |  |  |
| --- | --- | --- | --- | --- | --- | --- | --- | --- |
| Direction | Size | *T*  (mean) | *P* | *T* | Time  (ms) | Frequency | LR | Region |
| Positive | 161 | 2.699 | 0.046 | 3.313 | 3116 | Alpha | L | paracentral |
|  | 173 | 2.791 | 0.036 | 3.068 | 2124 | Delta | L | postcentral |
|  | 279 | 2.975 | 0.010 | 4.880 | 3492 | Low Gamma | L | precuneus |
|  | 313 | 3.109 | 0.008 | 5.022 | 3100 | Beta | L | superior parietal |
|  | 211 | 3.187 | 0.016 | 6.962 | 1656 | Beta | L | superior parietal |
|  | 213 | 2.476 | 0.026 | 3.139 | 916 | Delta | L | superior temporal |
|  | 160 | 2.792 | 0.036 | 3.266 | 1312 | Delta | L | temporal pole |
|  | 212 | 2.752 | 0.036 | 3.844 | 2960 | Alpha | R | superior parietal |
| Negative | 256 | -2.578 | 0.010 | -3.399 | 992 | Beta | L | bankssts |
|  | 280 | -3.230 | 0.020 | -4.546 | 932 | Theta | L | cuneus |
|  | 296 | -3.054 | 0.020 | -3.982 | 2748 | Theta | L | cuneus |
|  | 266 | -2.988 | 0.014 | -4.585 | 708 | Low Gamma | L | entorhinal |
|  | 195 | -3.668 | 0.018 | -4.542 | 2916 | Theta | L | entorhinal |
|  | 233 | -2.346 | 0.024 | -2.706 | 1936 | Theta | L | fusiform |
|  | 299 | -2.781 | 0.010 | -3.526 | 708 | Alpha | L | inferior parietal |
|  | 501 | -2.980 | 0.006 | -4.258 | 1368 | Theta | L | inferior temporal |
|  | 182 | -3.389 | 0.020 | -4.547 | 3088 | Theta | L | inferior temporal |
|  | 261 | -3.042 | 0.010 | -3.968 | 2700 | Theta | L | insula |
|  | 592 | -3.649 | 0.004 | -5.575 | 1288 | Theta | L | lateral occipital |
|  | 464 | -4.072 | 0.004 | -5.255 | 3360 | Theta | L | lateral occipital |
|  | 1221 | -3.234 | 0.002 | -5.640 | 884 | Theta | L | lingual |
|  | 187 | -3.411 | 0.020 | -5.342 | 72 | Theta | L | middle temporal |
|  | 208 | -2.597 | 0.032 | -3.072 | 3016 | Theta | L | middle temporal |
|  | 205 | -2.554 | 0.022 | -3.210 | 1092 | Theta | L | pars orbitalis |
|  | 1233 | -3.226 | 0.002 | -4.767 | 2688 | Theta | L | pericalcarine |
|  | 331 | -2.631 | 0.004 | -3.378 | 2240 | Theta | L | precuneus |
|  | 182 | -2.770 | 0.034 | -3.379 | 160 | Theta | L | precuneus |
|  | 938 | -3.246 | 0.010 | -4.537 | 728 | Beta | R | cuneus |
|  | 234 | -3.061 | 0.044 | -5.096 | 36 | Alpha | R | cuneus |
|  | 248 | -2.789 | 0.012 | -3.749 | 1592 | Theta | R | fusiform |
|  | 356 | -3.391 | 0.002 | -4.564 | 892 | Theta | R | inferior parietal |
|  | 276 | -3.364 | 0.008 | -4.596 | 1260 | Theta | R | inferior temporal |
|  | 151 | -2.914 | 0.042 | -3.487 | 2224 | Theta | R | inferior temporal |
|  | 146 | -2.866 | 0.044 | -3.515 | 140 | Theta | R | inferior temporal |
|  | 423 | -3.594 | 0.004 | -5.370 | 936 | Alpha | R | lateral occipital |
|  | 790 | -3.369 | 0.002 | -6.328 | 784 | Beta | R | lingual |
|  | 406 | -3.238 | 0.016 | -4.431 | 3188 | Theta | R | lingual |
|  | 218 | -2.834 | 0.018 | -3.808 | 108 | Theta | R | middle temporal |
|  | 207 | -2.897 | 0.018 | -3.721 | 1584 | Theta | R | middle temporal |
|  | 169 | -2.427 | 0.050 | -2.773 | 3452 | Theta | R | middle temporal |
|  | 507 | -3.026 | 0.002 | -4.005 | 1724 | Theta | R | parahippocampal |
|  | 188 | -3.169 | 0.034 | -3.724 | 812 | Theta | R | parahippocampal |
|  | 1300 | -2.958 | 0.002 | -5.373 | 720 | Beta | R | pericalcarine |
|  | 200 | -2.847 | 0.032 | -3.620 | 1044 | Theta | R | precuneus |
|  | 210 | -2.601 | 0.032 | -3.103 | 2768 | Delta | R | superior frontal |
|  | 202 | -2.412 | 0.034 | -2.798 | 328 | Delta | R | superior frontal |

*T* (mean), *T*-statistics averaged across data points included in each cluster; *P*, *P*-values; *T*, *T*-statistics.

**
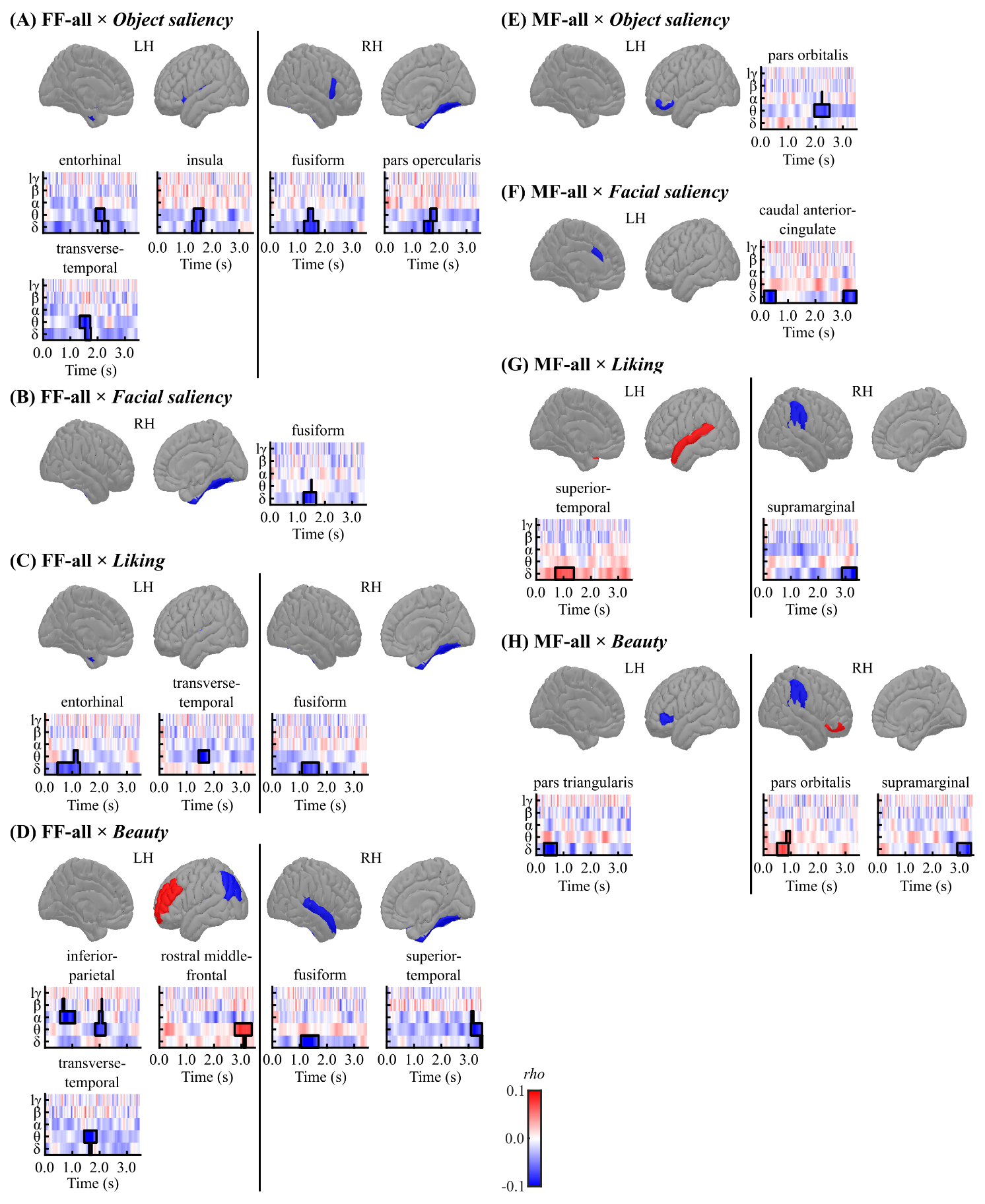
**

**Figure S8. (All data)** Results of the cluster-based permutation tests for evaluating correlations between regional TF data and pre-rating scores [**(A, E)** *object saliency*, **(B, F)** *facial saliency*, **(C, G)** *liking*, **(D, H)** *beauty*] in each condition [**(A**–**D)** FF-all and **(E**–**H)** MF-all] for **all data**. All TF images are scaled equally, with significant clusters emphasised using black bold lines. The regions (ROIs) in the template brain images were coloured red when any positive clusters were found in the ROI or blue when any negative clusters were found in the ROI. The results for hγ band are not displayed, because no significant clusters were found in the band. FF, face-face condition; MF, Mondrian-face condition; LH, left hemisphere; RH, right hemisphere; lγ, low-gamma; hγ, high-gamma.

**Table S9. (All data) Results of the cluster-based permutation tests for evaluating correlations between the TF data and pre-rating scores.**

|  |  | Cluster | | | Peak | | |  |  |
| --- | --- | --- | --- | --- | --- | --- | --- | --- | --- |
|  | Direction | Size | *T*  (mean) | *P* | *T* | Time  (ms) | Frequency | LR | Region |
| (A) FF-all × *Object saliency* | | | | | | | | | |
|  | Negative | 138 | -2.735 | 0.030 | -3.555 | 2216 | Delta | L | entorhinal |
|  |  | 170 | -2.611 | 0.014 | -3.393 | 1436 | Delta | L | insula |
|  |  | 151 | -3.049 | 0.024 | -4.440 | 1680 | Delta | L | transverse temporal |
|  |  | 179 | -2.694 | 0.010 | -3.431 | 1648 | Delta | R | fusiform |
|  |  | 138 | -2.512 | 0.040 | -3.135 | 1656 | Delta | R | pars opercularis |
| (B) FF-all × *Facial saliency* | | | | | | | | | |
|  | Negative | 115 | -3.328 | 0.032 | -4.194 | 1324 | Delta | R | fusiform |
| (C) FF-all × *Liking* | | | | | | | | | |
|  | Negative | 245 | -2.495 | 0.002 | -3.345 | 896 | Delta | L | entorhinal |
|  |  | 96 | -3.496 | 0.044 | -4.446 | 1644 | Theta | L | transverse temporal |
|  |  | 155 | -2.643 | 0.028 | -3.312 | 1260 | Delta | R | fusiform |
| (D) FF-all × *Beauty* | | | | | | | | | |
|  | Positive | 172 | 2.843 | 0.010 | 3.727 | 3164 | Theta | L | rostral middle frontal |
|  | Negative | 151 | -3.404 | 0.010 | -6.407 | 788 | Alpha | L | inferior parietal |
|  |  | 133 | -2.670 | 0.028 | -3.176 | 1932 | Theta | L | inferior parietal |
|  |  | 136 | -3.239 | 0.022 | -4.635 | 1736 | Theta | L | transverse temporal |
|  |  | 161 | -3.617 | 0.004 | -5.014 | 1264 | Delta | R | fusiform |
|  |  | 142 | -2.727 | 0.018 | -3.403 | 3500 | Delta | R | superior temporal |
| (E) MF-all × *Object saliency* | | | | | | | | | |
|  | Negative | 144 | -2.676 | 0.022 | -3.210 | 2340 | Theta | L | pars orbitalis |
| (F) MF-all × *Facial saliency* | | | | | | | | | |
|  | Negative | 117 | -2.994 | 0.020 | -3.612 | 3436 | Delta | L | caudal anterior cingulate |
|  |  | 106 | -2.942 | 0.038 | -4.424 | 452 | Delta | L | caudal anterior cingulate |
| (G) MF-all × *Liking* | | | | | | | | | |
|  | Positive | 172 | 2.486 | 0.024 | 2.902 | 748 | Delta | L | superior temporal |
|  | Negative | 135 | -2.799 | 0.036 | -3.448 | 3052 | Delta | R | supramarginal |
| (H) MF-all × *Beauty* | | | | | | | | | |
|  | Positive | 140 | 2.678 | 0.046 | 3.487 | 720 | Delta | R | pars orbitalis |
|  | Negative | 118 | -2.904 | 0.038 | -3.336 | 576 | Delta | L | pars triangularis |
|  |  | 126 | -2.502 | 0.050 | -2.800 | 3248 | Delta | R | supramarginal |

FF, face-face condition; MF, Mondrian-face condition; *T* (mean), *T*-statistics averaged across data points included in each cluster; *P*, *P*-values; *T*, *T*-statistics.
